# Gestational bisphenol A exposure induces fatty liver development in male offspring mice through the inhibition of HNF1b and upregulation of PPARγ

**DOI:** 10.1007/s10565-020-09535-3

**Published:** 2020-07-04

**Authors:** Zi Long, Junshu Fan, Guangyuan Wu, Xiyu Liu, Hao Wu, Jiangzheng Liu, Yao Chen, Shuhao Su, Xiaodong Cheng, Zhongrui Xu, Hongfei Su, Meng Cao, Chunping Zhang, Chunxu Hai, Xin Wang

**Affiliations:** 1 Department of Toxicology, Shaanxi Key Lab of Free Radical Biology and Medicine, the Ministry of Education Key Lab of Hazard Assessment and Control in Special Operational Environment, School of Public Health, Air Force Medical University (Fourth Military Medical University), Changle West Road 169, Xi’an, 710032 Shaanxi Province China; 2 Department of Biomedical Engineering, Air Force Medical University (Fourth Military Medical University), Xi’an, 710032 China

**Keywords:** BPA, Gestational exposure, HNF1b, PPARγ, Estrogen, Metabolism

## Abstract

Bisphenol A (BPA) is an endocrine-disrupting chemical (EDC) associated with non-alcoholic fatty liver disease (NAFLD). The effects of gestational BPA exposure on hepatic lipid accumulation in offspring are not fully understood. Here, we investigate the sex-dependent effects of gestational BPA exposure on hepatic lipid and glucose metabolism in the offspring of mice to reveal the mechanisms underlying gestational BPA exposure-associated NAFLD. Pregnant mice were administered gavage with or without 1 μg kg^−1^ day^−1^ BPA at embryonic day 7.5 (E7.5)–E16.5. Hepatic glucose and lipid metabolism were evaluated in these models. Both male and female offspring mice exhibited hepatic fatty liver after BPA treatment. Lipid accumulation and dysfunction of glucose metabolism were observed in male offspring. We revealed abnormal expression of lipid regulators in the liver and that inhibition of peroxisome proliferator-activated receptor γ (PPARγ) repressed hepatic lipid accumulation induced by gestational BPA exposure. We also found a sex-dependent decrease of hepatocyte nuclear factor 1b (HNF1b) expression in male offspring. The transcriptional repression of PPARγ by HNF1b was confirmed in L02 cells. Downregulation of HNF1b, upregulation of PPARγ, and subsequent upregulation of hepatic lipid accumulation were essential for NAFLD development in male offspring gestationally exposed to BPA as well as BPA-exposed adult male mice. Dysregulation of the HNF1b/PPARγ pathway may be involved in gestational BPA exposure-induced NAFLD in male offspring. These data provide new insights into the mechanism of gestational BPA exposure-associated sex-dependent glucose and lipid metabolic dysfunction.

**Electronic supplementary material:**

The online version of this article (10.1007/s10565-020-09535-3) contains supplementary material, which is available to authorized users.

**Graphical abstract:**
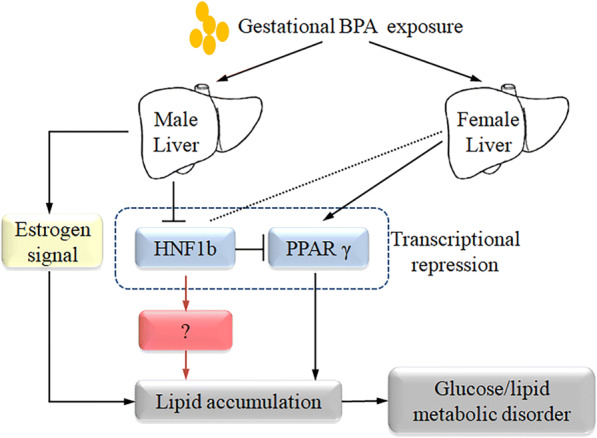
Schematic of the mechanism of gestational BPA exposure-induced glucose and lipid metabolic dysfunction.

## Introduction

The incidence of nonalcoholic fatty liver disease (NAFLD) has sharply increased since the 1990s. It is estimated that one billion people worldwide suffer from NAFLD with numbers expected to continuously increase (Loomba and Sanyal 2013). The causes of epidemic NAFLD currently remain unclear. Most studies have focused on high-calorie diets and unhealthy lifestyle; however, it is apparent that environmental factors also play a substantial role (Wang et al. 2013). Recent experimental, clinical, and epidemiological studies have shown that contaminants in food may negatively affect endocrine and metabolic functions, thereby promoting NAFLD and other related metabolic diseases (Marmugi et al. 2012). Prior to the increase of the incidence of NAFLD, synthetic chemicals being used in food has increased exponentially. These synthetic chemicals include BPA (IUPAC ID: 4,4′-(propane-2,2-diyl) diphenol) (Baillie-Hamilton 2002). BPA was first synthesized in 1891, and since 1957, has been incorporated as a component of plastic bottles (Vandenberg et al. 2007). Since then, BPA has become one of the most convenient and extensively used plasticizers in the manufacturing of polycarbonate plastics and epoxies.

Multiple studies have shown that continuous exposure to BPA can result in a variety of adverse health effects including sexual dysfunction, metabolic disorders, heart disease, and obesity (Bhandari et al. 2013; Dolinoy and Jirtle 2008; Hugo et al. 2008; Melzer et al. 2010; Li et al. 2010; Valentino et al. 2016; vom Saal and Myers 2008; vom Saal and Welshons 2014; Melzer et al. 2010). There are many evidence that BPA present in a large number of industrial products which inadvertently affects human health, through by ingestion of food or water. In addition to ingesting BPA, certain items such as airline tickets or thermal paper used in supermarkets, can be absorbed through the skin and can be an additional source of exposure to BPA (Biedermann et al. 2010; Ehrlich et al. 2014).

Contaminant exposure during pregnancy to environmental factors or EDCs could result in significant adverse outcomes in Nadal et al. (Nadal et al. 2014). In particular, gestational exposure to BPA induces a variety of pathophysiological conditions in offspring (Pu et al. 2017; Rahman et al. 2017; Vandenberg et al. 2019). However, there is no clear link between BPA exposure during pregnancy and metabolic disorders in offspring. BPA exposure pathways related to pregnancy and infants are difficult to define due to the diverse nature of BPA exposure. In addition, pathological phenomena including metabolic diseases, such as obesity and NAFLD, are complex and difficult to study (Lorenzo 2016; Roth et al. 2017; Stacy et al. 2016).

Studies on the effects of early BPA exposure on metabolic diseases are ambiguous. Early BPA exposure may increase or decrease the risk of obesity (Legeay and Faure 2017). Although there are some objections, relevant studies have shown a link between prenatal exposure to BPA and metabolic disorders, especially in respect to obesity and glycolipid homeostasis. According to epidemiological data, prenatal exposure to BPA can increase serum leptin levels (Rexford and Flier 2000), reduce serum adiponectin levels, reduce insulin sensitivity (Luo and Liu 2016), and lead to the occurrence of glucose and lipid metabolism disorders.

In this study, we investigate the effects of gestational BPA exposure on metabolic dysfunction in offspring of mice and reveal the mechanism underlying gestational BPA exposure-associated metabolic disorders. These mechanisms were unraveled with the use of in vitro tissue culture and in vivo mice through genetic manipulation and pharmacological intervention.

## Materials and methods

### Animal studies

Adult C57BL/6J mice were purchased from the Animal Centre of Air Force Medical University. All experiments were conducted in accordance with procedures approved by the Air Force Medical University Animal Care and Use Committee. Mice were housed at constant temperature (23 ± 2 °C), humidity (55 ± 5%), and strictly followed regular light cycles (12 h light/dark). In order to minimize background exposure to BPA beyond treatment regimen, polystyrene cages were used for feeding. A total of 10 male and 20 female mice were fed a normal diet (ND) (Medicience, Ltd. MD17212) and tap water ad libitum until 8 weeks of age. Then, mice were randomly divided into ten breeding pairs (male:female = 1:2). The following day, female mice were analyzed for vaginal plugs to confirm if mating occurred. Pregnant females were separated from males, and the date of vaginal plug observation was regarded as E0.5. Conceived mice were evenly divided into five groups: control, 1 μg kg^−1^ day^−1^ BPA, 10 μg kg^−1^ day^−1^ BPA, 100 μg kg^−1^ day^−1^ BPA, and 1000 μg kg^−1^ day^−1^ BPA. The pregnant mice were treated with corn oil or BPA at different concentrations through gavage. BPA administration was initiated at E7.5, before the development of the embryonic liver and was continued until E16.5. Offspring mice were separated from mothers at postnatal day (PND) 22 and were separated from one another after PND43. Mice were kept for another 8 weeks before being euthanized. After determining the optimal dose, 20 male and 40 female mice were randomly divided into 20 breeding pairs (male:female = 1:2). Pregnant mice were randomly divided into two equal groups. In group 1, mice were administered corn oil by gavage, and in group 2, mice were administered with 1 μg kg^−1^ day^−1^ BPA (Sigma) by gavage. BPA administration was initiated at E7.5, before the development of the embryonic liver, and resumed up until E16.5. Offspring mice were separated from females 3 weeks after delivery, and offspring mice were separated after an additional 3 weeks. After separation of offspring mice, different genders were randomly divided into five equal groups including a control group, high fat diet (HFD) group, BPA + HFD group, BPA + Rh1 (ginsenoside Rh1, inhibitor of PPARγ, MedChemExpress) group, and BPA group. Mice in the control, BPA + Rh1, and BPA groups were fed ND. Mice in the HFD and BPA + HFD groups were fed a diet containing 45% fat kcal% (Medicience, Ltd. MD12032) for 8 weeks. Ten weeks after birth, mice in the BPA + Rh1 group were orally administrated 20 mg kg^−1^ day^−1^ ginsenoside Rh1 (Tam et al. 2018) for 4 weeks. Fasted blood glucose levels were measured. Finally, the mice were anesthetized using 1% sodium pentobarbital after 8 h of fasting. After being killed, serum was collected, liver tissue were weighed, frozen sections of liver tissues were prepared, and tissues were frozen in liquid nitrogen before being stored at − 80 °C.

### Measurement of fasted blood glucose levels, intraperitoneal glucose tolerance test and intraperitoneal insulin tolerance test

Before euthanizing mice, fasted blood glucose levels were measured using a glucometer (SANNUO). Intraperitoneal glucose tolerance test (IPGTT) was used to evaluate the effect of glucose load on metabolic activity and intraperitoneal insulin tolerance test (IPITT) was performed to determine the extent of insulin resistance in mice. IPGTT and IPITT were performed 1 week prior to animals being euthanized, with a 3-day interval between the two experiments. After fasting for 8 h, basal levels of blood glucose were measured in tail blood samples of mice. Next, mice were intraperitoneally administered d-glucose (Sigma, 1 g kg^−1^ body weight) or insulin (Novolin R, 0.75 U kg^−1^ body weight). Blood glucose levels were measured at 30, 60, and 120 min post-injection.

### Cell culture

L02 cells (normal human liver cells) were purchased from the American Type Culture Collection (ATCC). L02 cells were cultured in high-glucose DMEM, phenol red free DMEM (PRF DMEM) medium (HyClone) containing 10% fetal bovine serum (FBS) or estrogen charcoal-stripped FBS (CS FBS), and 1% penicillin/streptomycin (100 μg ml^−1^) at a constant temperature (37 °C) in a humidified incubator (5% CO_2_). To ensure that the experimental results were not influenced by exogenous estrogen levels, PRF DMEM medium and CS FBS were used in our experiments that were conducted in preparation of CS FBS and was followed based on a prior work (de Faria et al. 2016; Simoncini et al. 2005). Fulvestrant (ICI 182780, MedChemExpress, 100 nM) was used for estrogen receptor inhibition. L02 cells were exposed to lentivirus (LV)-HNF1b (Genechem Co., Ltd., Shanghai) or LV-shPPARγ (TianYuCheng Co., Ltd., Xi’an) for 12 h and then selected with puromycin (BioFroxx, Germany) for 12 h to establish a cell line with stable overexpression of HNF1b. L02 cells were treated with 1 or 10 μM BPA for 48 h.

### Triglyceride analysis

Hepatic triglyceride (TG) levels were measured using a microplate fluorimeter (Infinite M200; Tecan, Hillsborough, NC) and Colorimetric Assay Kit (Beijing Solarbio Science & Technology, China).

### Tissue staining

Liver tissues were fixed in 4% paraformaldehyde solution and embedded in paraffin. Sections from paraffin-embedded tissue were stained with hematoxylin and eosin (H&E) following standard protocols. To detect lipid deposits in tissues, frozen sections were prepared and stained using Oil Red O (Beijing Solarbio Science & Technology, China) or BODIPY 493/503 (Life Technologies, USA). Results of H&E and Oil Red O staining were observed using a light microscope (Olympus) whereas BODIPY staining was observed using a laser scanning confocal microscope (LSCM) (Olympus).

### Glycogen content and PSA staining

A Glycogen Content Detection Kit (Beijing Solarbio Science & Technology, China) was used to detect glycogen content in cells and liver tissues according to the manufacturer’s protocol. Absorbance was measured using a microplate fluorimeter (Infinite M200; Tecan, Hillsborough, NC). A Glycogen Periodic Acid Schiff (PAS/Hematoxylin) Stain Kit (Beijing Solarbio Science & Technology, China) was used to display glycogen levels in liver tissue. PAS staining was observed using a light microscope (Olympus).

### Real-time-PCR

Total RNA of tissues and cells was isolated using a commercial RNA Isolation Assay Kit (TIANGEN, China). Next, 500 ng of target RNA was reversely transcribe into cDNA with the use of a TaKaRa PrimeScript RT-PCR Kit (TaKaRa, China). A total of 1 μl of cDNA was subjected to real-time (RT)-PCR in 20 μl reaction mixtures using a SYBR Green PCR kit (Thermo Scientific, USA) using a CFX96 real-time PCR system (BioRad, USA). PCR reactions were completed as follows: 95 °C for 10 min, then 95 °C for 1 min (30 cycles), annealing at 53 °C for 1 min, extension at 72 °C for 1 min, and final extension at 72 °C for 5 min. Relative amounts of mRNA levels were quantified using the comparative cycle threshold (C_T_) (2^−ΔΔ^CT) method. β-Actin was used as an internal control. Results were expressed as fold control. A list of primer sequences is included in both Tables S1 and S2.

### Western blotting

Total protein from tissues and cells was lysed using a RIPA lysis kit (TIANGEN, China). A BCA protein detection kit (Thermo Scientific, IL, USA) was used to determine protein concentrations that were used to adjust each sample to be the same protein concentration. Equal volumes of protein extraction (8–10 μl) and loading buffer were separated using a SDS-PAGE electrophoresis. Protein separated on the SDS-PAGE gel was transferred to a polyvinylidene fluoride (PVDF) membrane which was activated by methanol. After blocking in 10% evaporated milk for 2 h at 37 °C, membranes were incubated at 4 °C for 12–16 h with a specific primary antibody. Primary antibodies used include: GAPDH (Bioworld, AP0063 1:1000, ~ 36 kDa), PPARγ (Bioworld, BS1587, 1:1000, ~ 54 kDa), HNF1b (ProSci, Cat No. 55-640, 1:1000, ~ 61 kDa), ESR1 (Biorbyt, Cat. No. orb216104, 1:1000, ~ 66 kDa), ESR2 (Biorbyt, Cat. No. orb256527, 1:1000, ~ 60 kDa), PI3K (CST, Cat. No. 4255s, 1:1000, ~ 110 kDa), *p*-PI3K (CST, Cat. No. 17366s, 1:1000, ~ 85 kDa), Akt (CST, Cat. No. 4685s, 1:1000, ~ 60 kDa), *p*-Akt (CST, Cat. No. 4060s, 1:1000, ~ 60 kDa), and GLUT-4 (CST, Cat. No. 2213s, 1:1000, ~ 50 kDa). After washing in TBST for four times, membranes were incubated in HRP-conjugated secondary antibody (Bioworld, 1:4000) at 37 °C for 30 min. Protein bands were detected using a commercial chemiluminescent kit (Thermo, USA) and observed using a chemiluminescence image analyzer “Quantity One System” (Bio-Rad, USA). The quantification of protein expression was determined relative to of the endogenous loading control GADPH.

### Enzyme-linked immunosorbent assay

Serum was isolated by whole blood by centrifugation (2500 rpm, 15 min) after being placed at room temperature for 2–4 h before storing at − 20 °C. Insulin levels in serum were assayed using the ultrasensitive mouse insulin (INS) enzyme-linked immunosorbent (ELISA) kit (Cusabio Biotech Co., Ltd., Cat No. CSB-E05071m). Serum BPA levels were detected using a mouse BPA ELISA kit (JiangLai, Co., Ltd., JL16623). Serum estrogen receptor levels were detected using an estrogen receptor alpha (ERα) ELISA kit (ServiceBio, SEB050Mu).

### Transmission electron microscopy

Fresh liver tissues were collected and washed in phosphate-buffered solution (PBS). Prepared tissues were quickly placed in electron microscopy fixation solution (ServiceBio, G1102) and fixed at 4 ° for 2–4 h. Next, tissues were fixed in 1% osmium tetroxide for 2 h at room temperature and washed three times in PBS. Fixed liver tissue was dehydrated using a gradient of alcohol and embedded with 812 embedding agent (SPI, 90529-77-4). Finally, ultrathin sections were prepared and stained in 2% uranium acetate solution and lead citrate for 15 min. After the sample was prepared, the ultrastructure of hepatocytes was observed and photographed using a transmission electron microscope (Hitachi, HT7700).

### Real time cell analysis

xCELLigence real-time cell analysis (RTCA) S16 (ACEA Biosciences) was used to dynamically monitor cell proliferation. A total of 100 μl of medium was added to each well of an E-plate 16 to calibrate the data to the standard value before adding 50 μl of 1 × 10^5^ ml^−1^ cell suspension to each well. The program started to be monitored after the plate sat for 30 min.

### Chromatin immunoprecipitation

L02 cells were treated with or without 10 μM BPA for 48 h. In order to assess the binding of HNF1b in the promoters of PPARγ, a chromatin immunoprecipitation assay kit (Thermo Scientific, USA) was used according to manufacturer’s instructions. First, the cells were cross-linked with formaldehyde, incubated at 37 °C for 10 min, then the cross-linking was terminated, the cells were broken by ultrasonication, impurities were removed by centrifugation, and the cross-linking was unwound. Antibodies of HNF1b and corresponding IgG were used for incubation, immune complexes were washed, and DNA was recovered. Subsequently, detailed RT-PCR assays were performed and the relative mRNA levels were quantified using the comparative cycle threshold (CT) (^2−ΔΔ^CT) method. IgG binding was used as a negative control. Primer sequences are included in Table S2.

### Statistical analysis

Data was expressed as mean ± SEM. Student’s *t* tests were used to assess statistically significant differences between two groups. One-way analysis of variance (ANOVA) followed by the Newman-Keuls multiple-comparison post hoc test was used to analyze differences between more than two groups. All data were analyzed using GraphPad Prism 7.0 (GraphPad Software, Inc. La Jolla, CA, USA) and were considered statistically significant if *p* < 0.05.

## Results

### Low-dose BPA exposure during pregnancy causes an accumulation in liver TGs

**Fig. 1:**
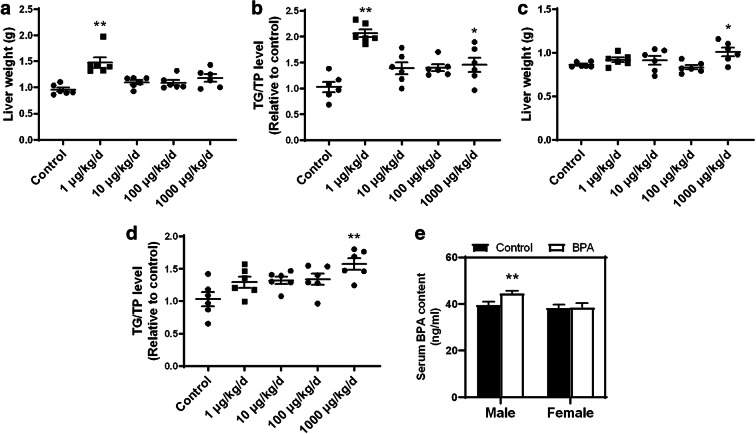
Effects of prenatal exposure to different concentrations of BPA on hepatic lipid accumulation. **a**, **c** Livers of male and female offspring were weighted, and **b**, **d** TG content was determined. Serum BPA content was measured using ELISA (**e**). *p < 0.05; **p < 0.01, indicate significant differences when compared with respective controls

To determine the optimal dose of gestational BPA exposure, four different doses including 1, 10, 100, and 1000 μg kg^−1^ day^−1^ were selected to treat pregnant mice with the same exposure window and measured liver weight and liver TG content. In male offspring, a significant increase in liver weight at 1 μg kg^−1^ day^−1^ was observed when compared with the control group. However, with increased concentration of BPA that was administered, the liver weight did not significantly change (Fig. 1a). A similar trend was shown for TG content, but the TG content only slightly increased at 1000 μg kg^−1^ day^−1^ (Fig. 1b). Inverse results were shown for female offspring. The liver weight of female offspring showed a slight difference at 1000 μg kg^−1^ day^−1^, similar to what was shown for TG content (Fig. 1c, d). Since low-dose BPA exposure is closer to what humans are exposed to in real-life settings, a BPA dose of 1 μg kg^−1^ day^−1^ was used for the remaining experiments of this study. In addition, we tested the BPA content in the serum of mice exposed to 1 μg kg^−1^ day^−1^ BPA and found that this content increased in the serum of male mice but showed no significant changes in female mice (Fig. 1e). Furthermore, we confirmed that the BPA content in mouse serum was very close to BPA levels observed in humans.

### Gestational exposure to BPA affects glucose and lipid metabolism in male offspring

**Fig. 2:**
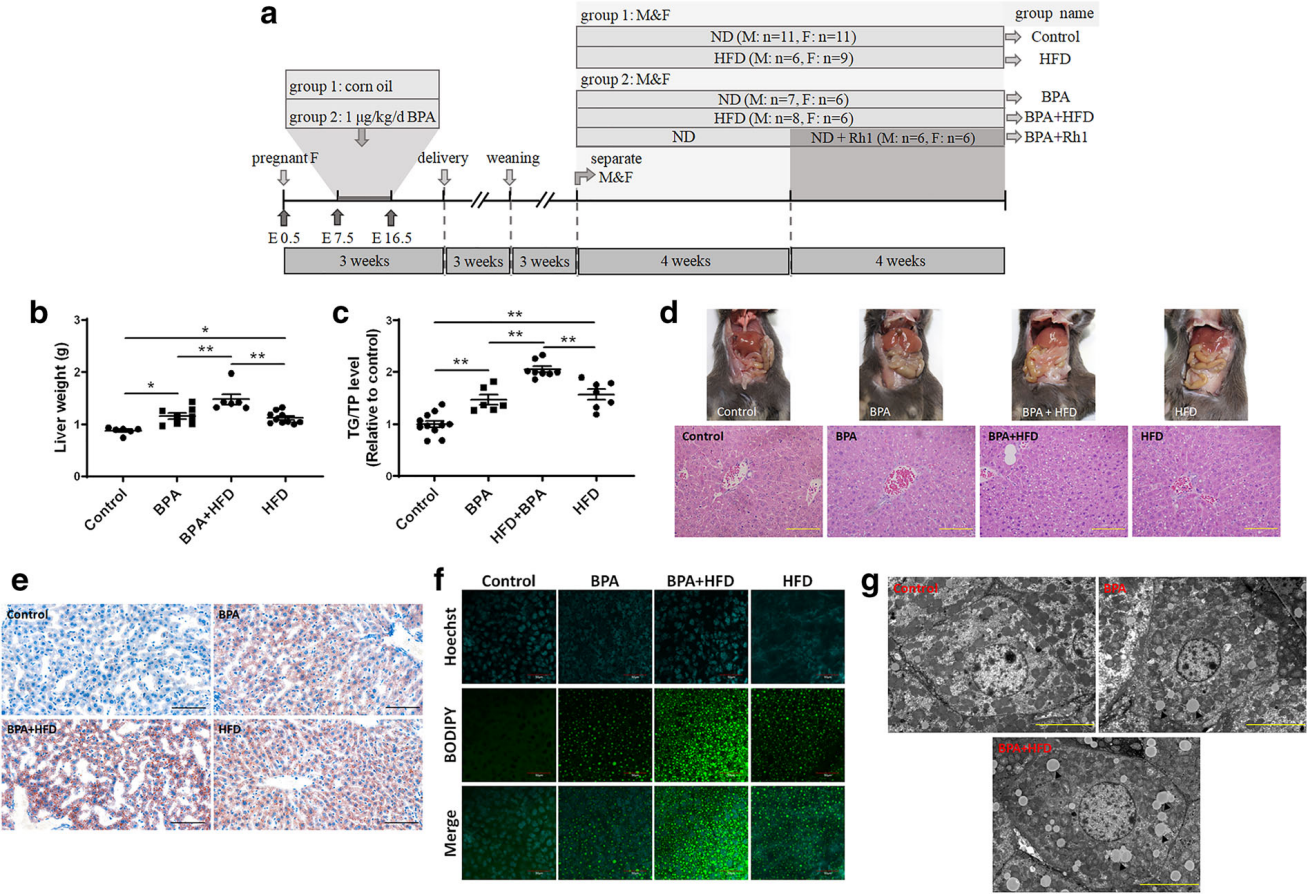
Effects of gestational BPA exposure on hepatic lipid accumulation in male offspring. **a** The animal treatment procedure conducted is shown in a schematic. **b** Liver weight as well as **c** TG content were determined. *p < 0.05; **p < 0.01, indicate significant differences when compared between the two groups. **d** H&E staining of liver tissue is shown. Frozen sections were using (**e**) Oil Red O and (**f**) BODIPY to observe lipid droplets. Size bar, 50 μm. **g** Electron microscopy was used to observe intracellular lipid corpuscles. Size bar, 2 μm

To investigate the effects of gestational BPA exposure on hepatic lipid accumulation in offspring, pregnant mice were exposed to 1 μg kg^−1^ BPA during E7.5–E16.5. The 6-week-old offspring that was or was not exposed to BPA was also given either ND or a combination containing HFD for 8 weeks, with the goal of observing lipid accumulation in the liver (Fig. 2a). Surprisingly, it appeared that gestational exposure to BPA resulted in a sex-dependent effect on hepatic lipid accumulation in adult male offspring. As shown in Fig. 2b, c, BPA induced liver weight gain and increased hepatic TG levels in male offspring reaching adulthood and was even worse when exposed to HFD. Moreover, BPA markedly increased the number of liver lipid droplets induced by HFD (Fig. 2d–f). Electron microscopy also revealed an increase in the number of intracellular lipid corpuscles observed in BPA-exposed liver tissue (Fig. 2g).

**Fig. 3:**
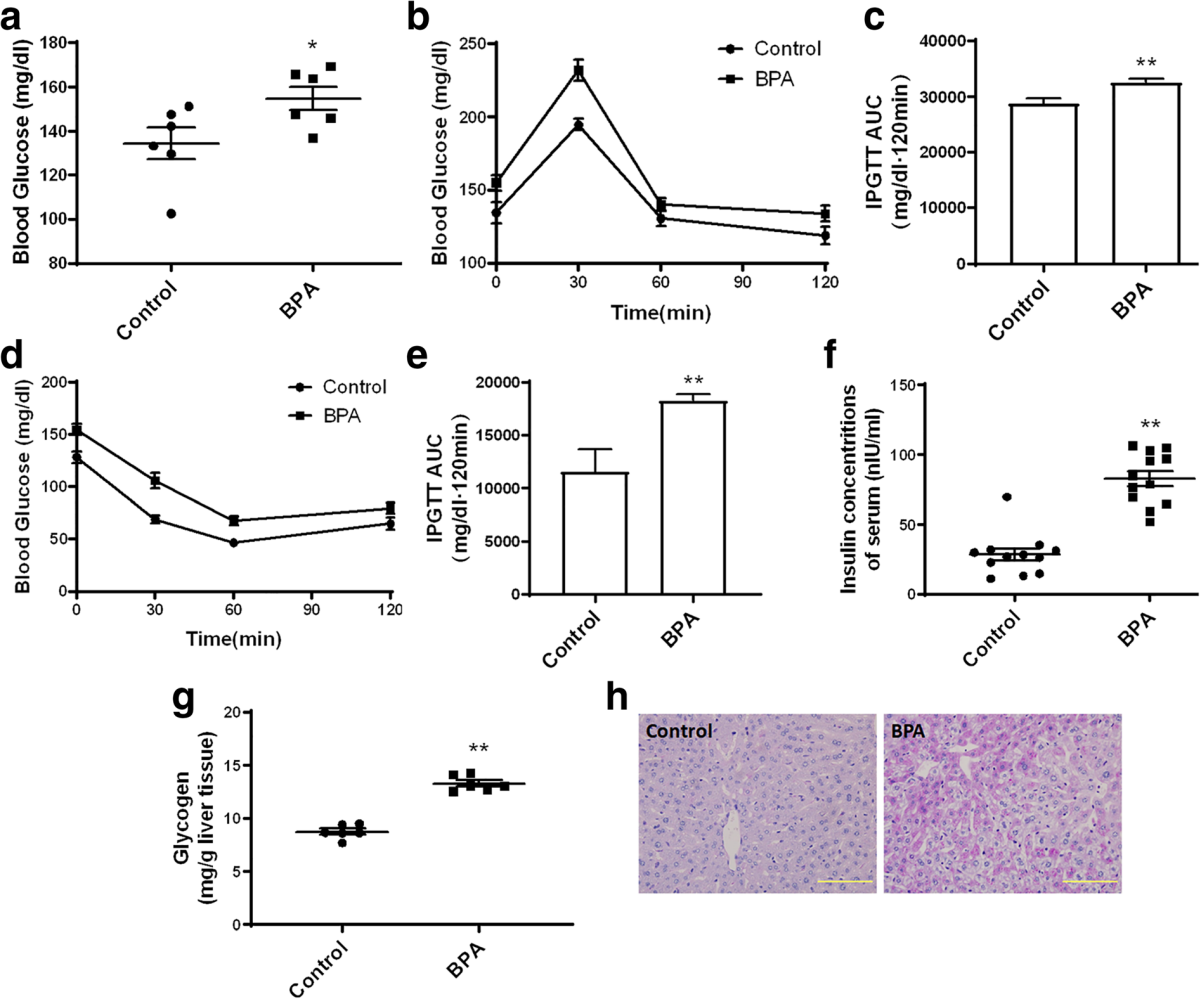
Effects of gestational BPA exposure on glucose metabolism in male offspring. **a** Fasting blood glucose levels were determined using a glucometer. One week prior to anesthetization, IPGTT was conducted to evaluate **b** glucose tolerance activity and **c** to calculate the area under the curve. IPITT was conducted to evaluate **d** insulin tolerance activity, and **e** AUC was calculated. **f** Serum insulin content was measured using ELISA. **g** Glycogen content was evaluated, and **h** PSA staining was performed for liver tissues. Size bar, 50 μm; *p < 0.05; **p < 0.01, indicate significant differences when compared with respective controls

Considering the fundamental role that abnormal lipid metabolism plays in the dysfunction of glucose metabolism, the effects of gestational BPA exposure on glucose metabolism in adult male offspring were next investigated. BPA administration leads to a significant increase in the levels of fasting blood glucose (Fig. 3a). It was also found that the blood glucose levels in IPGTT and IPITT tests were markedly increased by BPA exposure, as illustrated by a significant increase in the area under the curves (Fig. 3b–e). Serum insulin levels were also significantly elevated in the BPA-treated group compared with the BPA-untreated group (Fig. 3f). Quantitative analysis and PSA staining revealed that glycogen content in the liver of BPA-exposed mice was significantly increased compared with what? (Fig. 3g, h). These results indicate that gestational exposure to BPA causes significant deregulation of glucose and lipid metabolism in male offspring when they reach adulthood.

### Gestational exposure to BPA affects glucose and lipid metabolism in female offspring

**Fig. 4:**
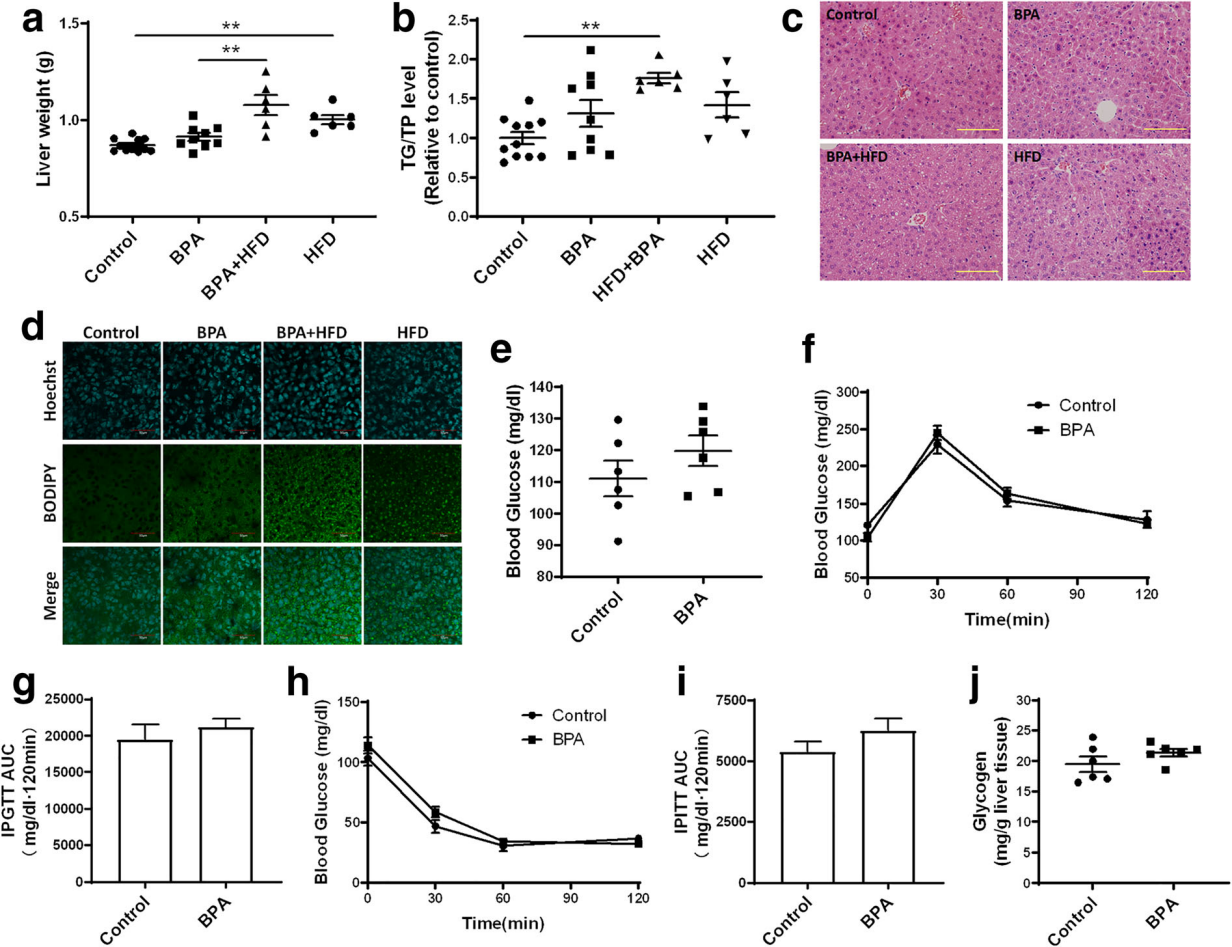
Effects of gestational BPA exposure on lipid accumulation and glucose metabolism in female offspring. **a** Liver weight was determined, and **b** TG content was also determined to assess lipid accumulation in liver tissue. H&E staining of liver tissue was performed to observe **c** histological changes. **d** Frozen sections were stained with BODIPY to observe lipid droplets. Size bar, 50 μm. **e** Fasting blood glucose levels were determined using a glucometer. One week prior to the anesthetization, IPGTT was conducted to evaluate **f** glucose tolerance activity as well as **g** the area under the curve. IPITT was conducted to evaluate **h** insulin tolerance activity and to calculate **i** AUC. **j** Glycogen content was evaluated in liver tissues. Size bar, 50 μm; **p < 0.01, indicate significant differences when compared between the two groups

A similar phenomenon was observed in female mice; however, there were no significant effects of BPA on liver weight gain or hepatic TG levels in these offspring (Fig. 4a, b). The degrees of glucose metabolism and lipid droplet accumulation were less pronounced than what was observed in the male group (Fig. 4c, d). Administration of BPA did not significantly increase fasting glucose levels (Fig. 4e). IPGTT and IPITT blood glucose levels and the areas under the curve did not show significant changes after BPA exposure, (Fig. 4f–i). Quantitative analysis of glycogen content in the liver showed no significant changes in BPA-exposed mice (Fig. 4j). These results suggest that gestational exposure to BPA did not impair glucose and lipid metabolism in adult female offspring. Although both male and female offspring exhibited glucose and lipid metabolism disorders induced by BPA treatment, lipid accumulation and glucose metabolism dysfunction were significantly different in the male offspring only. Thus, subsequent experiments focused on studies involving male offspring only.

### Exposure to BPA affects glucose and lipid metabolism

Since it was found that gestational exposure to BPA induces hepatic lipid accumulation and metabolic dysfunction in male offspring, we next tested whether direct exposure to BPA affected adult male mice and cultured liver cells, similarly. Adult male mice were exposed to 1 μg kg^−1^ day^−1^ BPA for 8 weeks to identify the molecular mechanisms behind f BPA-induced effects. As shown in Supplemental Fig. 1a, BPA increased hepatic TG levels in male adult mice and facilitated the HFD-induced increase of hepatic TG levels. BPA markedly increased the number of liver lipid droplets induced by HFD (Supplemental Fig. 1b–d). Furthermore, quantitative analysis and PSA staining revealed that glycogen content in the liver of BPA-exposed mice was significantly increased (Supplemental Fig. 1e, f). Similar to male mice offspring exposure to BPA during gestation, significant glucose and lipid metabolic changes were observed in adult male mice exposed to BPA, which was further aggravated by HFD-induced effects.

We next sought to see whether similar trends existed in vitro. To exclude the effects of exogenous estrogen, trace levels were removed from culture media and serum. In Supplemental Fig. 2a, RTCA was used to detect cell proliferation to find that the use of PRF medium did not significantly affected cell growth. Even though CS FBS reduced cell growth rate, it did not affect cell state. In vitro assays using L02 liver cells revealed a BPA concentration-dependent increase in the degree of fatty acid uptake, the absorbance of Oil Red O staining and glycogen content (Supplemental Fig. 2b–d) as well as lipid accumulation (Supplemental Fig. 2h), and decreased insulin-stimulated glucose uptake (Supplemental Fig. 2e). These data indicate that there is an occurrence of dysfunction in glucose and lipid metabolism. A concentration of 10 μM BPA exhibited the strongest effects and was selected for subsequent in vitro experiments. L02 cells were incubated with PA for 24 h to stimulate lipid accumulation. BPA increased fatty acid uptake and aggravated PA-induced increase in fatty acid uptake in L02 cells (Supplemental Fig. 2f). Moreover, BPA markedly increased lipid accumulation in L02 cells treated with PA (Supplemental Fig. 2g, h). Quantitative analyses revealed that glycogen content in L02 cells that were exposed to BPA was significantly increased (Supplemental Fig. 2i). These results demonstrated that in vitro cell line exposure to BPA not only promotes lipid accumulation and glucose metabolic dysfunction in L02 cells but also aggravates PA-induced glucose and lipid metabolic abnormalities.

### Gestational exposure to BPA affects the expression of key lipid metabolism regulator genes

**Fig. 5:**
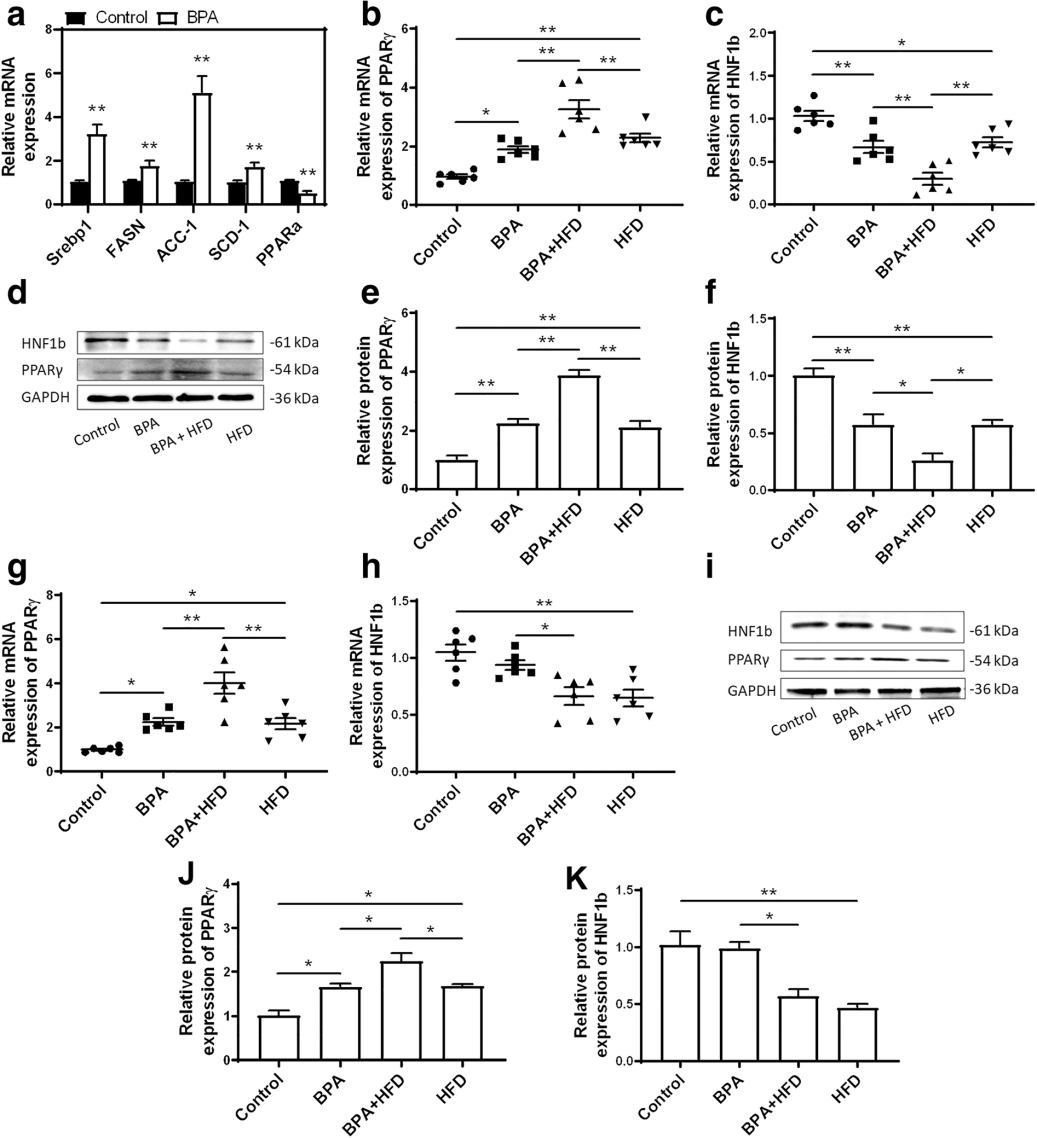
Effects of gestational BPA exposure on the expression of key regulators involved in in vivo lipid metabolism. Genes involved in lipid metabolism regulation in the liver were determined in male offspring. **a** These genes include *Srebp1*, *FASN*, *ACC-1*, *SCD-1*, and *PPARα*; **p < 0.01, indicate significant differences when compared with respective controls. The mRNA and protein expression levels of *PPARγ* and *HNF1b* were determined in the liver tissues of **b**–**f** male and **g**–**k** female offspring using qRT-PCR and immunoblotting. *p < 0.05; **p < 0.01, indicate significant differences when compared between the two groups

To explore the mechanisms behind BPA-induced glucose and lipid metabolic disorders, expression levels of genes involved in the regulation of lipid metabolism were interrogated. Exposure to BPA during pregnancy can increases mRNA expression levels of various regulators of lipogenesis that are involved in de novo fatty acid synthesis (fatty acid synthase (FASN), acetyl-CoA carboxylase (ACC-1), sterol regulatory element binding protein-1 (Srebp1)) and triglyceride synthesis (stearoyl-coenzyme A desaturase 1 (SCD-1)) (Fig. 5a). BPA can also decrease the peroxisome proliferator activated receptor α (PPARα) expression, which is responsible for fatty acid oxidation (Fig. 5a). BPA exposure leads to a significant increase in both mRNA and protein expression levels of PPARγ in the livers of mice. In contrast, the mRNA and protein expression levels of HNF1b are markedly decreased in mouse livers exposed to BPA. Additionally, BPA and HFD together result in an even greater increase in the mRNA and protein expression levels of PPARγ. In contrast, BPA significantly reduced both mRNA and protein expression levels of HNF1b in HFD-treated mice (Fig. 5b–f). These data reveal that gestational exposure to BPA does not only promote lipid absorption and synthesis in the liver but also aggravates HFD-induced lipid metabolism abnormalities.

**Fig. 6:**
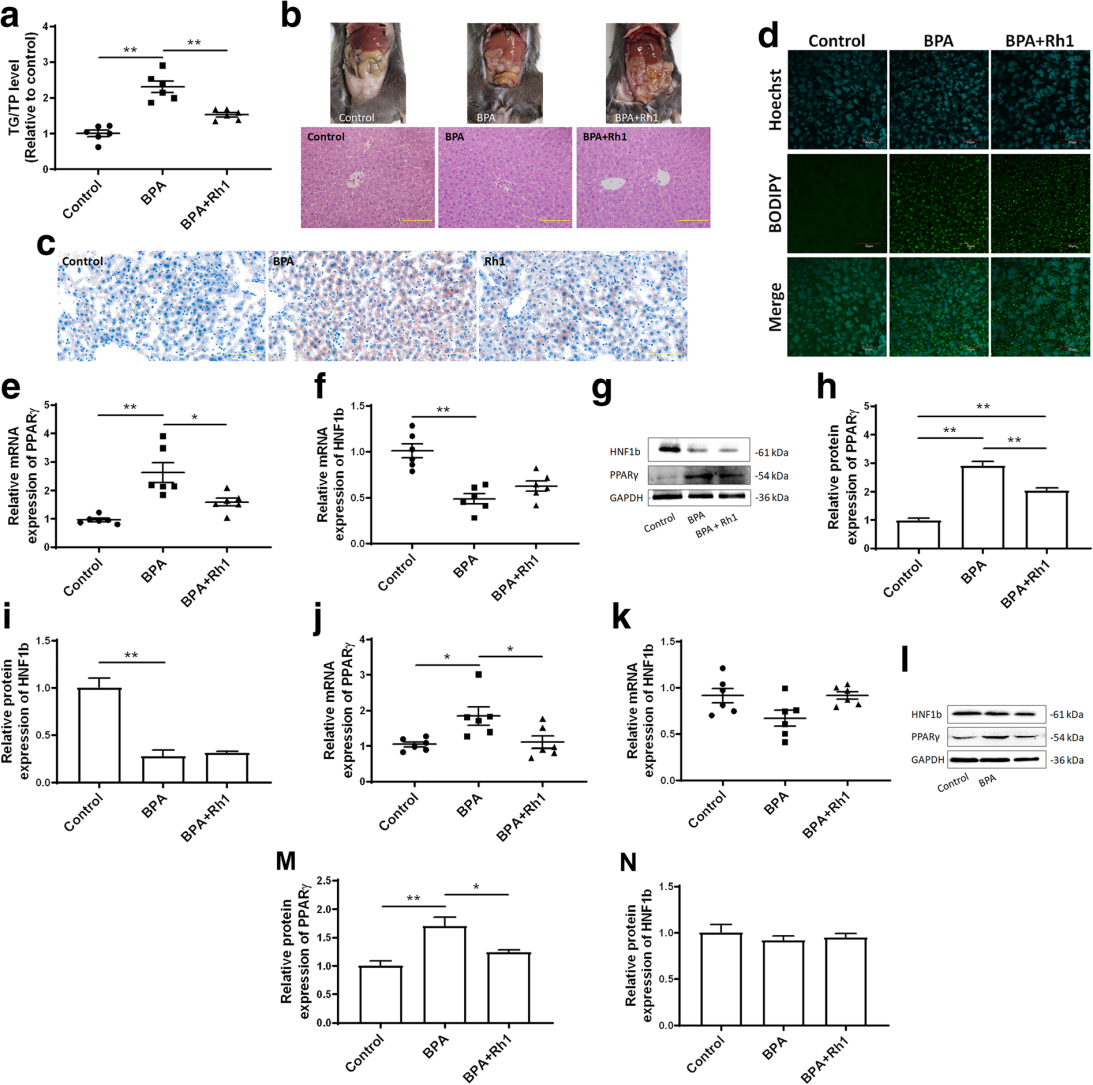
Upregulation of PPARγ is involved in gestational BPA exposure-induced effects in male offspring. Determination of **a** liver TG content and **h** H&E staining of liver tissues are shown. Frozen sections were stained using (**c** Oil Red O and **d** BODIPY to observe lipid droplets. Size bar, 50 μm. Both mRNA and protein expression levels of *PPARγ* and *HNF1b* were determined in the liver tissues of (**e**–**i**) male and (**j**–**n**) female mice using qRT-PCR and immunoblotting. *p < 0.05; **p < 0.01, indicate significant differences when compared between the two groups

In order to confirm whether PPARγ plays a key role in BPA-induced metabolic dysfunction in the liver, male offspring mice exposed to BPA gestationally were treated with ginsenoside Rh1, a specific inhibitor of PPARγ, for 4 weeks at 10-week age. In ginsenoside Rh1-treated mice, the BPA-induced increase of TG content normally seen in BPA-treated mice alone was significantly inhibited (Fig. 6a). In addition, in ginsenoside Rh1-treated mice, there was a significant reduction of hepatic lipid droplets and lipid accumulation was improved (Fig. 6b–d). Moreover, the BPA-induced increase of PPARγ expression was inhibited by ginsenoside Rh1 (Fig. 6e, g, h). However, the BPA-induced decrease of HNF1b expression was not significantly altered by ginsenoside Rh1 (Fig. 6f, g, i), implicating that HNF1b was not regulated by PPARγ signaling. Altogether, these results suggest that upregulation of PPARγ is involved in hepatic lipid accumulation that is induced by gestational exposure to BPA.

The expression levels of PPARγ and HNF1b were also determined in the livers of female offspring mice gestationally exposed to BPA. We found that mRNA and protein expression levels of PPARγ were increased in the livers of female mice. However, there were no significant effects of BPA exposure observed on the mRNA and protein expression levels of HNF1b in female offspring (Figs. 5g–k and 6j–n). HNF1b-induced regulation of specific signaling pathways other than PPARγ signaling may be more critical and play more prominent roles in male offspring.

In addition, we detected molecules associated with the phosphoinositol 3 kinase (PI3K)/Akt (protein kinase B (PKB)) signaling pathway. The results showed that the phosphorylation of PI3K and Akt was reduced (Supplemental Fig. 3a–d). And we found that mRNA and protein expression levels of glucose transporter-4 (GLUT-4) were decreased in the livers of gestational BPA-exposed male mice (Supplemental Fig. 3e–g). This suggests that BPA did not activate the PI3K/Akt signaling pathway.

### Adult mice exposure to BPA affects the expression of key glucose and lipid metabolism regulators

In vivo and in vitro BPA exposure can alter the expression levels of various regulators of lipogenesis (Supplemental Fig. 4). In vivo*,* The mRNA expression levels of Srebp1, FASN, and SCD-1 were significantly increased by BPA exposure, and the mRNA expression levels of ACC-1 and PPARα were decreased by BPA (Supplemental Fig. 4a). Similarly, in vitro mRNA expression levels of Srebp1, FASN, ACC-1, and SCD-1 were significantly increased in response to BPA exposure, whereas the mRNA expression levels of PPARα were decreased (Supplemental Fig. 4b). There was a significant increase in the mRNA and protein expression levels of PPARγ induced by BPA both in vivo and in vitro. However, the mRNA and protein expression levels of HNF1b were markedly downregulated. BPA exposure aggravated the HFD and PA-induced increase of the mRNA and protein expression levels of PPARγ both in vivo and in vitro, respectively. In contrast, BPA significantly reduced both mRNA and protein expression levels of HNF1b in the livers of HFD-treated mice and PA-treated L02 cells (Supplemental Fig. 4c–f). These results demonstrated that the dysregulation of lipogenesis-associated regulators may be the culprit behind BPA-induced effects as well as BPA-induced aggravation along with HFD.

To confirm whether PPARγ plays a key role in BPA-induced metabolic dysfunction in the liver, 12-week-old male mice exposed to BPA were treated with ginsenoside Rh1 for 4 weeks. In ginsenoside Rh1-treated mice, the BPA-induced increase of TG content was significantly inhibited (Supplemental Fig. 5a). In addition, hepatic lipid droplets were significantly reduced and lipid accumulation was improved by ginsenoside Rh1 (Supplemental Fig. 5b–d). As shown in Supplemental Fig. 5e, f, quantitative analysis and PSA staining revealed that glycogen content in the liver of BPA-exposed mice was significantly reduced by ginsenoside Rh1 treatment. Moreover, the BPA-induced increase of PPARγ expression was inhibited by ginsenoside Rh1 (Supplemental Fig. 5g), whereas HNF1b expression was not significantly affected (Supplemental Fig. 5h). This implicates that HNF1b is not regulated by PPARγ signaling. Altogether, these results suggest that the BPA-induced upregulation of PPARγ is involved in hepatic lipid accumulation and glucose metabolic dysfunction both in vivo and in vitro.

### PPARγ regulate BPA-induced glucose and lipid metabolism through a different pathway of estrogen

We next tested estrogen-related indicators to determine whether estrogen plays a role in the development of glucose and lipid metabolism disorders in adult offspring mice exposed to BPA during gestation. ELISA was used to measure the amount of estrogen receptor 1 (ESR1) in the serum of male and female mice, and it was found that the levels of ESR1 in the serum of the BPA-exposed group was increased in male mice, with no significant differences found in female mice (Supplemental Fig. 6a). In addition, the mRNA and protein expression levels of ESR1 and estrogen receptor 2 (ESR2) in both male and female mice were measured. Also, the mRNA expression levels in L02 liver cells were measured. Estrogen receptor expression significantly increased in male mice (Supplemental Fig. 6b–d) whereas only ESR1 increased in females (Supplemental Fig. 6e–g). In vitro data were similar to those of which were observed in male mice exposed to BPA (Supplemental Fig. 6h). We also analyzed the expression of a series of estrogen-related transcription factors to find similar trends that were observed for estrogen receptor expression. The expression levels of perilipin 2 (Plin2), acetyl-CoA carboxylase 1 (ACACA), uncoupling protein 2 (Ucp2), and CCAAT/enhancer binding protein alpha (C/EBPα) in male offspring and L02 cells were increased. These trends were not shown in female mice besides Plin2 which showed similar results as observed in male mice (Supplemental Fig. 6i–k).

**Fig. 7:**
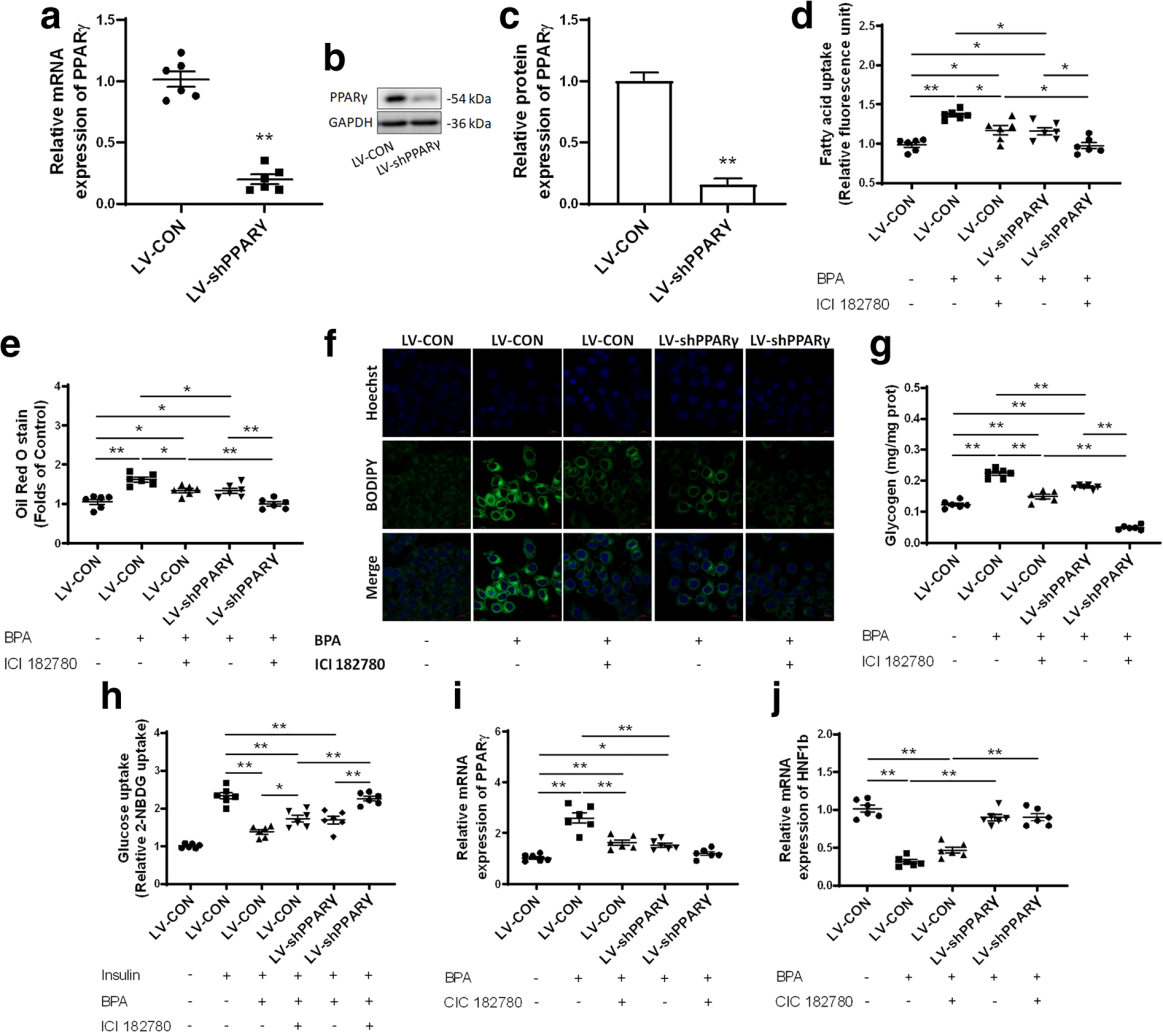
Both estrogen and PPARγ regulate BPA-induced glucose and lipid metabolism in vitro. **a**–**c** Both mRNA expression and protein expression of *PPARγ* were determined in LV-shPPARγ. **p < 0.01, indicate significant differences when compared with respective controls. **d** Fatty acid uptake was analyzed as well as **e** Oil Red O staining and **f** BODIPY staining were both performed to observe lipid accumulation in L02 cells. **g** Glycogen content and **h** glucose uptake were determined to evaluate dysfunction in glucose metabolism. **i**, **j** The mRNA expression levels of *PPARγ* and *HNF1b* were determined in L02 cells using qRT-PCR and immunoblotting. *p < 0.05; **p < 0.01, indicate significant differences when compared between the two groups

L02 liver cells were next transfected with LV-shPPARγ in combination with ICI 182780. In LV-shPPARγ-transfected cells, the expression levels of PPARγ were significantly reduced (Fig. 7a–c). As a result, both knockdown of PPARγ alone as well as ICI 182780 alone can significantly alleviate BPA-induced lipid accumulation and glucose metabolism abnormalities, but neither can completely reverse the metabolic disorders. The combination of LV-shPPARγ and ICI 182780 together almost completely restored BPA-induced glucose and lipid metabolism disorders (Fig. 7d–h). Furthermore, the BPA-induced increase of PPARγ expression was inhibited by LV-shPPARγ and ICI 182780 showed no effect on PPARγ expression (Fig. 7i). Nevertheless, the BPA-induced decrease of HNF1b expression was not significantly affected by LV-shPPARγ and ICI 182780 (Fig. 7j), implicating that HNF1b was not regulated by PPARγ signaling.

### BPA inhibits HNF1b-induced transcriptional suppression of PPARγ

**Fig. 8:**
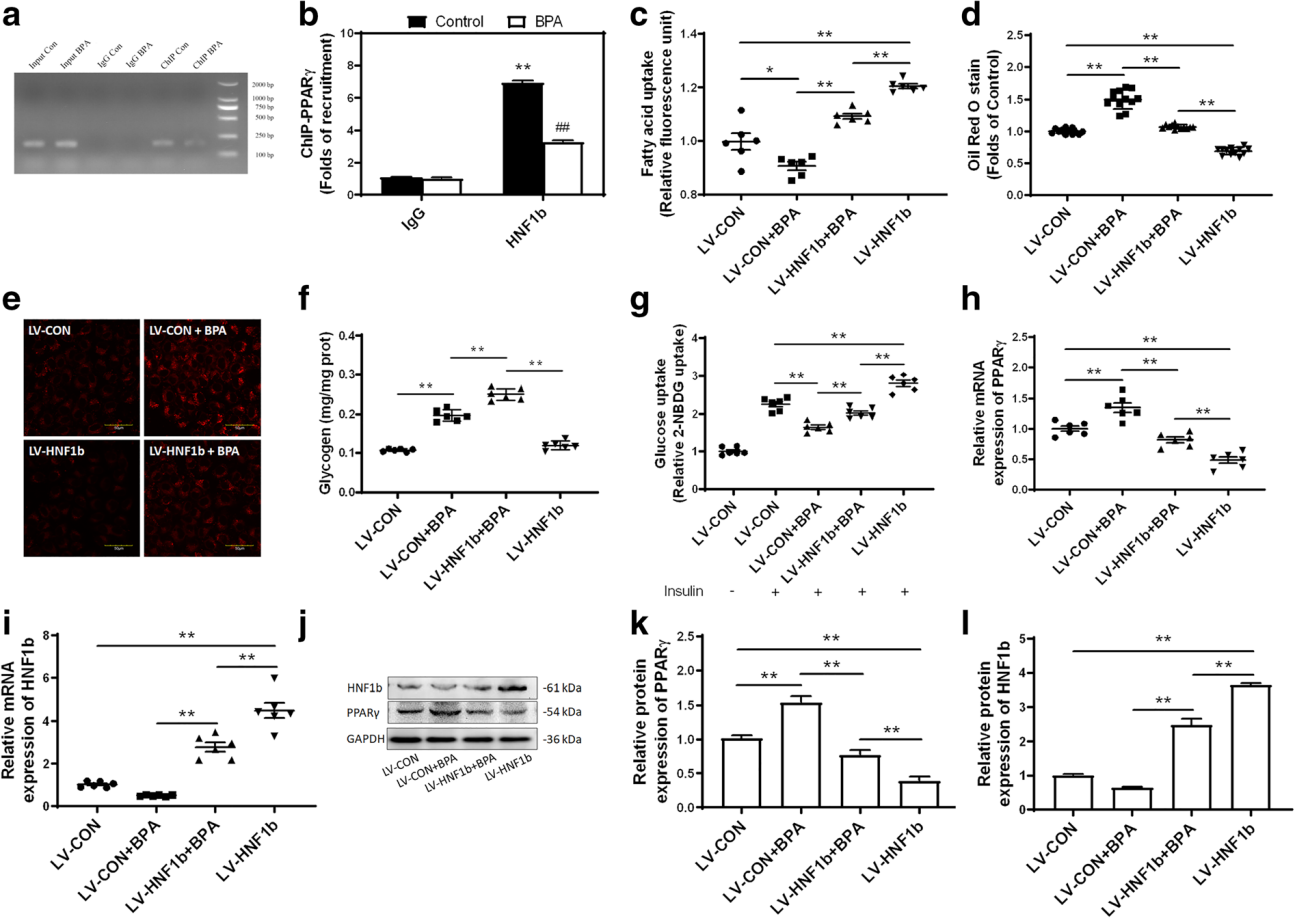
The downregulation of HNF1b is involved in BPA-induced glucose/lipid metabolic dysfunction. **a**, **b** HNF1b binding on the promoters of PPARγ in L02 cells was detected using ChIP, **p < 0.01 indicates significant differences when compared with control, and ^##^*p* < 0.01 indicates significant differences when compared with BPA. **c** Fatty acid uptake was analyzed as well as **d** Oil Rred O staining and **e** BODIPY staining were performed to observe lipid accumulation in L02 cells. **f** Glycogen content and **g** glucose uptake were both analyzed in LV-transfected L02 cells to evaluate dysfunction in glucose metabolism. **h**–**l** Both mRNA and protein expression levels of *PPARγ* and *HNF1b* were determined using qRT-PCR and immunoblotting. *p < 0.05; **p < 0.01, indicate significant differences when compared between the two groups

In order to understand the interaction between HNF1b and PPARγ, we performed a chromatin immunoprecipitation (ChIP) assay to assess HNF1b binding to PPARγ promoters under steady-state conditions. As shown in Fig. 8a, b, BPA significantly decreased HNF1b binding to PPARγ promoters. In order to detect whether HNF1b was involved in BPA-induced glucose and lipid metabolic dysfunction, L02 cells were transfected by LV-HNF1b to establish a stable cell line with the overexpression of HNF1b. Overexpression of HNF1b was confirmed through RT-PCR. Overexpression of HNF1b in LV-transfected L02 liver cells reduced the BPA-induced increase of fatty acid uptake (Fig. 8c, d) and Oil Red O staining (Fig. 8e) as well as prominently increased insulin-stimulated glucose uptake (Fig. 8f) and glycogen content (Fig. 8g), suggesting that overexpression of HNF1b alleviated BPA-induced glucose and lipid metabolic dysfunction. The BPA-induced increase of mRNA and protein expression levels of PPARγ was significantly inhibited by the upregulation of HNF1b (Fig. 8h–l). These results demonstrated that HNF1b functioned as a transcriptional repressor of PPARγ expression and that the dysregulation of HNF1b and PPARγ played a role in the BPA-induced increase of hepatic lipid accumulation and glucose metabolic dysfunction.

## Discussion

BPA is an EDC that mimics the effects of estrogen (Vandenberg et al. 2007) and has been shown to promote puberty (Howdeshell et al. 1999), reduce fertility (Cabaton et al. 2011; Salian et al. 2009), influence the male and female ratio of tadpoles and fish (Tompsett et al. 2012; Levy et al. 2004), induce obesity (Manikkam et al. 2013), as well as result in metabolic diseases (vom Saal and Myers 2008), hepatic steatosis (Martella et al. 2016), and cancers (Prins et al. 2007). BPA is found in many products including plastic bottles, medical and dental devices, thermal paper (Vandenberg et al. 2007; Halden 2010), sealants for dental fillers, optical discs and electronics, eyeglass lenses, and the lining of water pipes and tanks (Talsness et al. 2009; Huang et al. 2012). There is increasing evidence of widespread human exposure to BPA, which is detected in human plasma, urine, and breast milk (Vandenberg et al. 2012; Welshons et al. 2006; Calafat et al. 2008).

To understand the effects of BPA on health in humans, we established a mouse model to assess the effect of BPA perinatal exposure on metabolic disorders. The highest level of BPA detected in human maternal plasma is 82.8 nM and 40 nM in fetal plasma (Schönfelder et al. 2002). Since 2015, the European Food Safety Agency (EFSA) has modified the maximum tolerable daily intake (TDI) of BPA from 50 to 4 g kg^−1^ day^−1^ (Legeay and Faure 2017). Based on epidemiological data, current BPA safety limits and our experimental results, pregnant females were administered either 0 or 1 μg kg^−1^ day^−1^ BPA by gavage. That concentration was similar to the BPA content in the plasma of pregnant human women. In order to determine the window of exposure for BPA, we referred to other studies (Liu et al. 2013) which describe significant differences regarding how exposure windows affect body weight and glucose homeostasis. It is well known that mouse-fertilized eggs implant to the uterus at E5, whereas mouse fetal livers mature at E15–18 (Murakami-Kawaguchi et al. 2014; Rosen et al. 2009; Izzotti et al. 2003; Salimi et al. 2019). Genomics studies (Lee et al. 2012) showed that E16.5 is the dividing line between mature and immature liver development. Thus, E7.5–E16.5 was selected as the window of the BPA pregnancy exposure model for this study.

Other work has shown that BPA causes significant changes in normal metabolism. In one study (Manukyan et al. 2019), pregnant rats administered 0.5 μg kg^−1^ day^−1^ of BPA in water from E3.5 to PND22 showed that stimulated (11 mM glucose) insulin secretion was enhanced by 50% in islets isolated from BPA-exposed female and male offspring from dams. In other work, perinatal exposure to BPA was significantly associated with higher plasma TG concentrations and iWAT adipocyte density (Lejonklou et al. 2017) as well as a promotion of insulin resistance (Dunder et al. 2018). Another study (Shimpi et al. 2017) treated CD-1 rats with o 25 μg kg^−1^ day^−1^ of BPA from E8-PND16 and proved that exposure to BPA during early development can induce sustained fat accumulation by regulating hypomethylation of lipid-related genes. In addition, another study (Somm et al. 2009) showed that SD rats were given 1 mg l^−1^ BPA in drinking water from E6 through the end of lactation, which increased adipogenesis in females at weaning. Compared with other studies, a dose that is five times lower than the current TDI was chosen as well as a shorter and more specific exposure window was used to verify whether exposure to BPA during pregnancy had damaging effects on offspring mice.

It is generally believed that exposure to adverse factors after birth impacts obesity and metabolic disease development, as food intake and metabolic circuits of the hypothalamus are primarily established at this stage. Thus, the development of these important circuits is primarily affected by BPA neonatal exposure, which is not the only mechanism by which it can cause metabolic disorders. Numerous studies have shown that gestational BPA exposure also played an important role in adult metabolic disorders. The precise timing of the leptin surge after birth is critical for food intake and normal development of metabolic neural circuits (Bouret et al. 2015). Leptin secretion peaks are delayed in perinatal mice exposed to BPA (MacKay et al. 2017). Interruption of leptin surge time impairs the development of the melanocortin system, leading to changes in the hypothalamic feeding circuit, which may lead to metabolic disorders in adulthood. The key-deregulated genes associated with BPA exposure were found to be involved in de novo fatty acid synthesis and triglyceride synthesis. These data are consistent with earlier studies (Marmugi et al. 2014; Neuschwander-Tetri 2010), which revealed the major cause in BPA-induced disorders is aberrant lipid synthesis.

Our data revealed significant gender differences. The exact mechanism of gestational BPA exposure-induced sex-dependent fatty liver development is still not clear. In this study, we revealed that gestational BPA exposure resulted in a more severe fatty liver in male offspring and identified that HNF1b was differentially regulated in male and female offspring. We proposed that differential regulation of HNF1b may be an important reason for the sex-dependent effects of gestational exposure to BPA. The differential regulation of PPARγ and HNF1b might be due to the sex-dependent effect of gestational BPA exposure on hepatic lipid accumulation. Further studies are needed to clarify the mechanism of gestational BPA-induced differential regulation of HNF1b in different genders.

PPARγ is a key controller of lipid absorption and synthesis. It is well known that PPARγ can induce adipocyte differentiation and promote adipogenesis, increasing insulin sensitivity and improving NAFLD (Tang and Lane 2012). The mechanism by which BPA may affect adipogenesis is unclear, but it is possible that PPARγ may play a role (Chamorro-Garcia et al. 2012; Phrakonkham et al. 2008; Riu et al. 2011; Sargis et al. 2010; Somm et al. 2009). Previous studies have shown that prenatal exposure to BPA increases PPARγ expression in the liver of offspring mice (Garcia-Arevalo et al. 2014). Here, we observed elevated levels of PPARγ in the livers of fetuses exposed to BPA that at the same time experienced insulin resistance. To further understand this observation, we examined the expression of the PI3K/Akt signaling pathway and found that elevated PPARγ expression did not activate PI3K and Akt. Moreover, we also analyzed GLUT-4 expression levels to find that it was downregulated. These data indicate that the increase in PPARγ expression in gestational BPA-exposed offspring did not activate the PI3K/Akt signaling pathway. This may be one of the causes of BPA-induced insulin resistance.

Our data reveal that both the PPARγ and estrogen receptor pathways play important roles in BPA-induced glucose and lipid metabolism disorders, but their modes of action are relatively independent. Knockdown of PPARγ can alleviate the disorder of glucose and lipid metabolism induced by BPA, but it cannot make the phenomenon of lipid accumulation and glucose metabolic abnormalities disappear. Similarly, the use of estrogen inhibitors alone cannot reverse the occurrence of BPA-induced glucose and lipid metabolism disorders. But the combined effect of suppressing these two signal pathways can nearly eliminate damages resulting from BPA exposure. It shows that PPARγ does not respond to BPA exposure through the estrogen signaling but exerts the damaging effect through other ways.

HNF1b, also known as TCF2, LF-B3, vHNF1, and HNF1β, is a liver-enriched transcription factor of the homeodomain-containing superfamily that is highly conserved across species (Tronche and Yaniv 1992). Loss of function or a truncated HNF1b allele can result in maturity onset diabetes of the young, type 5 (MODY5), which is an autosomal dominant disorder (Bell et al. 1991; Edghill et al. 2006; Edghill et al. 2008; Bingham and Hattersley 2004; Chantelot et al. 2005), characterized by early onset usually 17–25.8 years old and is often accompanied by genital malformation. Moreover, a large population-based cohort study showed that genetic risk variability of HNF1b was significantly associated with the granule composition of lipoproteins (Stancáková et al. 2011). Our previous studies have shown that HNF1b plays an important role in regulating liver fat synthesis, adipocyte differentiation, glucose homeostasis, and insulin resistance (Long et al. 2017; Wang et al. 2017). Our results are consistent with previous studies revealing that HNF1b promotes glucose uptake and glycolytic activity, which was shown in ovarian clear carcinoma cells (Okamoto et al. 2015). HNF1b is a target of mir-802 in the development of obesity-associated impairment of glucose metabolism (Kornfeld et al. 2013). Our previous studies showed that upregulation of HNF1b could reduce the key regulators of de novo fatty acid synthesis and fatty acid extension (Quan et al. 2014), such as Srebp1, ACC-1, and FASN, as well as decrease TG content and increase insulin-dependent glucose uptake in liver cells (Wu et al. 2017). In combination with previous findings, our results demonstrated that HNF1b functioned as a regulator through controlling PPARγ expression. The downregulation of HNF1b/PPARγ played a large role in BPA-induced glucose and lipid metabolic dysregulation observed in the male offspring of female mice exposed to BPA during pregnancy.

Our study has some limitations and several questions still need to be answered. (1) The results in the study suggest that putative downstream targets of HNF1b, other than PPARγ, may play an important role in the observations witnessed in this study. More effort should be spent on the search for new targets that are responsible for the HNF1b-induced regulation of fatty acid synthesis, which may affect the response of offspring to gestational BPA exposure. (2) Our results show that the mice are more sensitive to low-dose BPA exposure than higher doses, in the context of fatty liver development. This raises new questions as to whether exposure to higher doses of BPA produces the same pathological effects and whether its regulatory mechanisms are the same as BPA exposure in adult mice. (3) Since hepatic HNF1b in male and female offspring is differentially regulated by BPA, we propose that differential mechanisms, such as epigenetic regulation of HNF1b/PPARγ signaling may exist. Moreover, although the role of HNF1b dysregulation was verified in vitro, mice where HNF1b is genetically manipulated should be used to confirm the role of HNF1b in the context of gestational exposure.

In summary, we identified that gestational BPA exposure affects a series of lipid-metabolism-related genes, leading to a sex-dependent hepatic lipid accumulation in offspring. Inhibition of PPARγ can significantly ameliorate BPA-induced glucose and lipid metabolic disorders in both male offspring mice and adult mice dysregulation of HNF1b may be involved in lipid accumulation and glucose metabolism deregulation in male offspring exposed to BPA during fetal development, through transcriptional repression of PPARγ (Graphical abstract). These data provide new insight into the mechanisms of gestational BPA-induced sex-dependent glucose and lipid metabolism deregulation as well as highlight HNF1b/PPARγ signaling in the regulation of lipid and glucose metabolism.

## Electronic supplementary material

ESM 1 (DOCX 4438 kb)

## Data Availability

Data will be available on request.
