# Supplementary material for: Gestational bisphenol A exposure induces fatty liver development in male offspring mice through the inhibition of HNF1b and upregulation of PPARγ
Source: Cell Biol Toxicol. 2020 Jul 4;37(1):65–84. doi: 10.1007/s10565-020-09535-3 (PMC7851022; doi:10.1007/s10565-020-09535-3)
Supplement: Supplementary file 1 — (DOCX 4438 kb) [file 10565_2020_9535_MOESM1_ESM.docx]

**Table S1 Primers (mouse) used in the study.**

| Gene | Forward | Reverse |
| --- | --- | --- |
| β-actin | 5’-AGATCATTGCTCCTCCTGAGCGCA-3’ | 5’-AAACGCAGCTCAGTAACAGTCCGC-3’ |
| Srebp1 | 5’-GCAGCCACCATCTAGCCTG-3’ | 5’-CAGCAGTGAGTCTGCCTTGAT-3’ |
| FASN | 5’-GGAGGTGGTGATAGCCGGTAT-3’ | 5’-TGGGTAATCCATAGAGCCCAG-3’ |
| ACC-1 | 5’-GATGAACCATCTCCGTTGGC-3’ | 5’-GACCCAATTATGAATCGGGAGTG-3’ |
| SCD-1 | 5’-TTCTTGCGATACACTCTGGTGC-3’ | 5’-CGGGATTGAATGTTCTTGTCGT-3’ |
| PPAR α | 5’-AGAGCCCCATCTGTCCTCTC-3’ | 5’-ACTGGTAGTCTGCAAAACCAAA-3’ |
| PPAR γ | 5’-CTCTGTTTTATGCTGTTATGGGTGA-3’ | 5’-GGTCAACAGGAGAATCTCCCAG-3’ |
| HNF1b | 5′-CGACGACTATGACACTCC-3′ | 5′-TGTTGCATGTATCCCTTG-3′ |
| ESR1 | 5′-CATAACAGCCTCGGAACGGA-3′ | 5′-TTTCACTCAACCAGCAGCCA-3′ |
| ESR2 | 5′-GCCTTACTTCCCCTGCTTCT-3′ | 5′-TCCAAGGGTAGGATGGACTG-3′ |
| Ucp2 | 5′-AAAGCAGCCTCCAGAACTCC-3′ | 5′-AAAGCAGCCTCCAGAACTCC-3′ |
| Plin2 | 5′-TGAGTGGCCTGTGTTAGTCT-3′ | 5′-TGAGTGGCCTGTGTTAGTCT-3′ |
| ACACA | 5′-GCATGTCTGGCTTGCACCTA-3′ | 5′-GCATGTCTGGCTTGCACCTA-3′ |
| C/EBPα | 5′-GAGACCGAGAGACTTTCCGC-3′ | 5′-GAGACCGAGAGACTTTCCGC-3′ |

**Table S2 Primers (human) used in the study.**

| Gene | Forward | Reverse |
| --- | --- | --- |
| β-actin | 5’-AGAGCTACGAGCTGCCTGAC-3’ | 5’-GGATGCCACAGGACTGGA-3’ |
| Srebp1 | 5’-GCGGAGCCATGGATTGCAC-3’ | 5’-CTCTTCCTTGATACCAGGCCC-3’ |
| FASN | 5’-AGCTGCCAGAGTCGGAGAAC-3’ | 5’-TGTAGCCCACGAGTGTCTCG-3’ |
| ACC-1 | 5’-TCGCTTTGGGGGAAATAAAGTG-3’ | 5’-ACCACCTACGGATAGACCGC-3’ |
| SCD-1 | 5’-CTTCTTGCGATACACTCTGG-3’ | 5’-TGAATGTTCTTGTCGTAGGG-3’ |
| PPAR α | 5’-CCAGTATTTAGGAAGCTGTCC-3’ | 5’-TGAAAGCGTGTCCGTGAT-3’ |
| PPAR γ | 5’-TCTGGCCCACCAACTTTGGG-3’ | 5’-CTTCACAAGCATGAACTCCA-3’ |
| HNF1b | 5’-GGCAATTGCACAAATGTCCTCT-3’ | 5’-ATTGTCTGAGGTGCCAGCAG-3’ |
| ESR1 | 5’-CCAGCACCCTGAAGTCTCTG-3’ | 5’-CCAGCACCCTGAAGTCTCTG-3’ |
| ESR2 | 5’-ACCACAAGCCCAAATGTGTT-3’ | 5’-GCGATGGACCACTAAAGGAG-3’ |
| Ucp2 | 5’-AGTCCGGTTACAGATCCAAGG-3’ | 5’-AGTCCGGTTACAGATCCAAGG-3’ |
| Plin2 | 5’-TGATGGCAGGCGACATCTAC-3’ | 5’-TGATGGCAGGCGACATCTAC-3’ |
| ACACA | 5’-TTGCCACCCTGAGGTCTTTTT-3’ | 5’-TGGTTCAGCTCCAGAGGTTG-3’ |
| C/EBPα | 5’-GGACCCTCAGCCTTGTTTGT-3’ | 5’-GGACCCTCAGCCTTGTTTGT-3’ |
| ChIP primers | 5'-GTTGTCTGAGTCCCTCGGTGT-3' | 5'-TTTCGGGTCCCTTGGTTTT-3' |

**Supplemental Fig.1. Effects of BPA on lipid accumulation *in vivo*.**


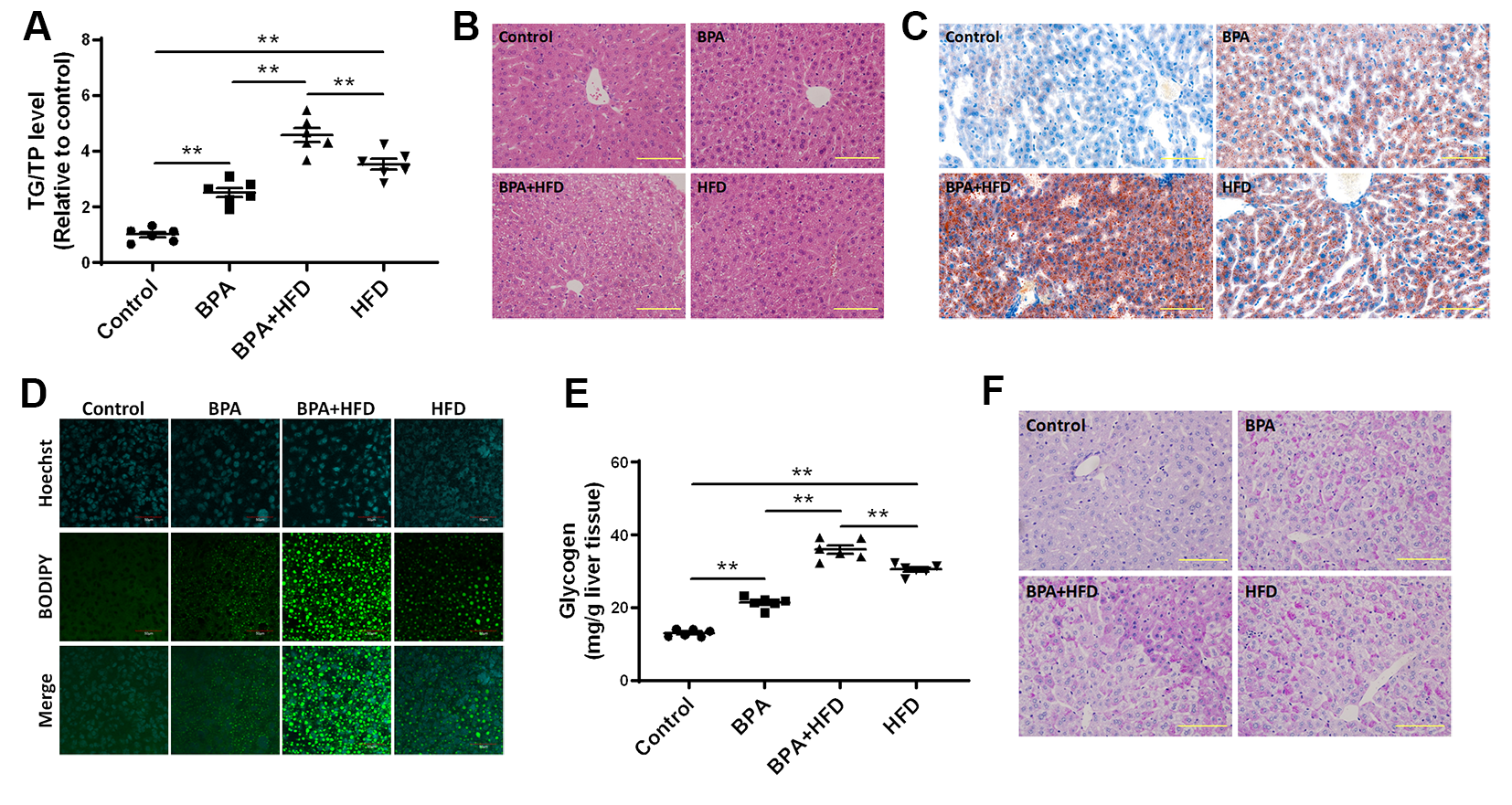


Determination of Liver TG contents in adult male mice. (A) H&E staining was performed to assess lipid accumulation in liver tissue and to (B) observe histological changes. Frozen sections were prepared and stained using (C) Oil Red O and (D) BODIPY to observe lipid droplets in liver tissues. (E) Glycogen content was detected and (F) PSA staining was used to display glycogen in liver tissues. Size bar =50μm. **p < 0.01, indicate significant differences when compared between the two groups.


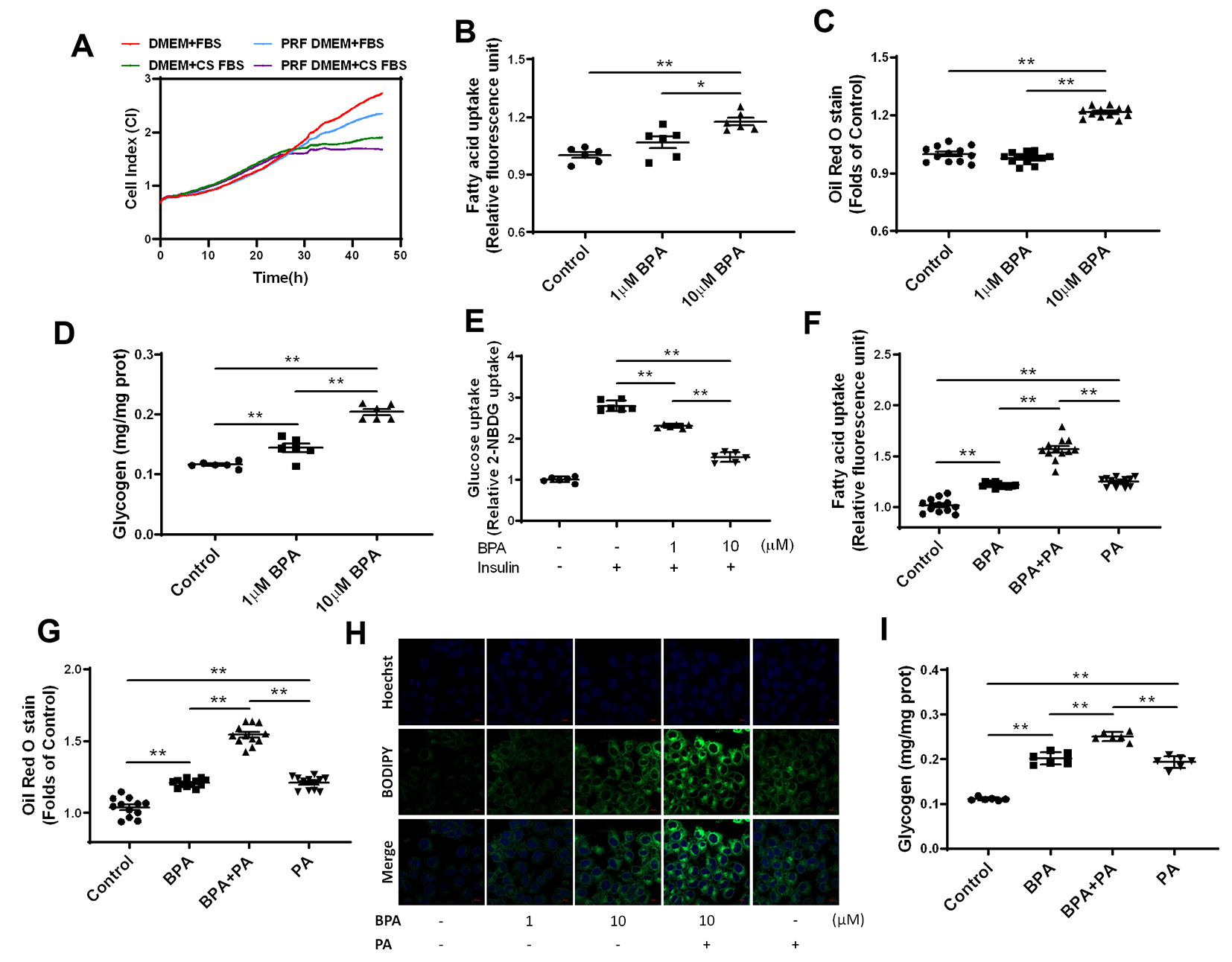


**Supplemental Fig.2. BPA induces glucose/lipid metabolic dysfunction *in vitro*.**

Cell index were measured using (A) RTCA. L02 cells were treated with 1 μM and 10 μM BPA. (B) Fatty acid uptake was determined and (C) Oil Red O staining was performed to observe lipid accumulation. (D) Glycogen content and (E) glucose uptake were both determined to evaluate dysfunction of glucose metabolism. Next, L02 cells were exposed to 10 μM BPA with or without 20μM of PA. (F) A fatty acid uptake assay, (G) Oil Red O staining and (H) BODIPY staining were performed to observe lipid accumulation. (I) glycogen content levels were determined in L02 cells. Size bar =50μm. *p < 0.05, **p < 0.01, indicate significant differences when compared between the two groups.


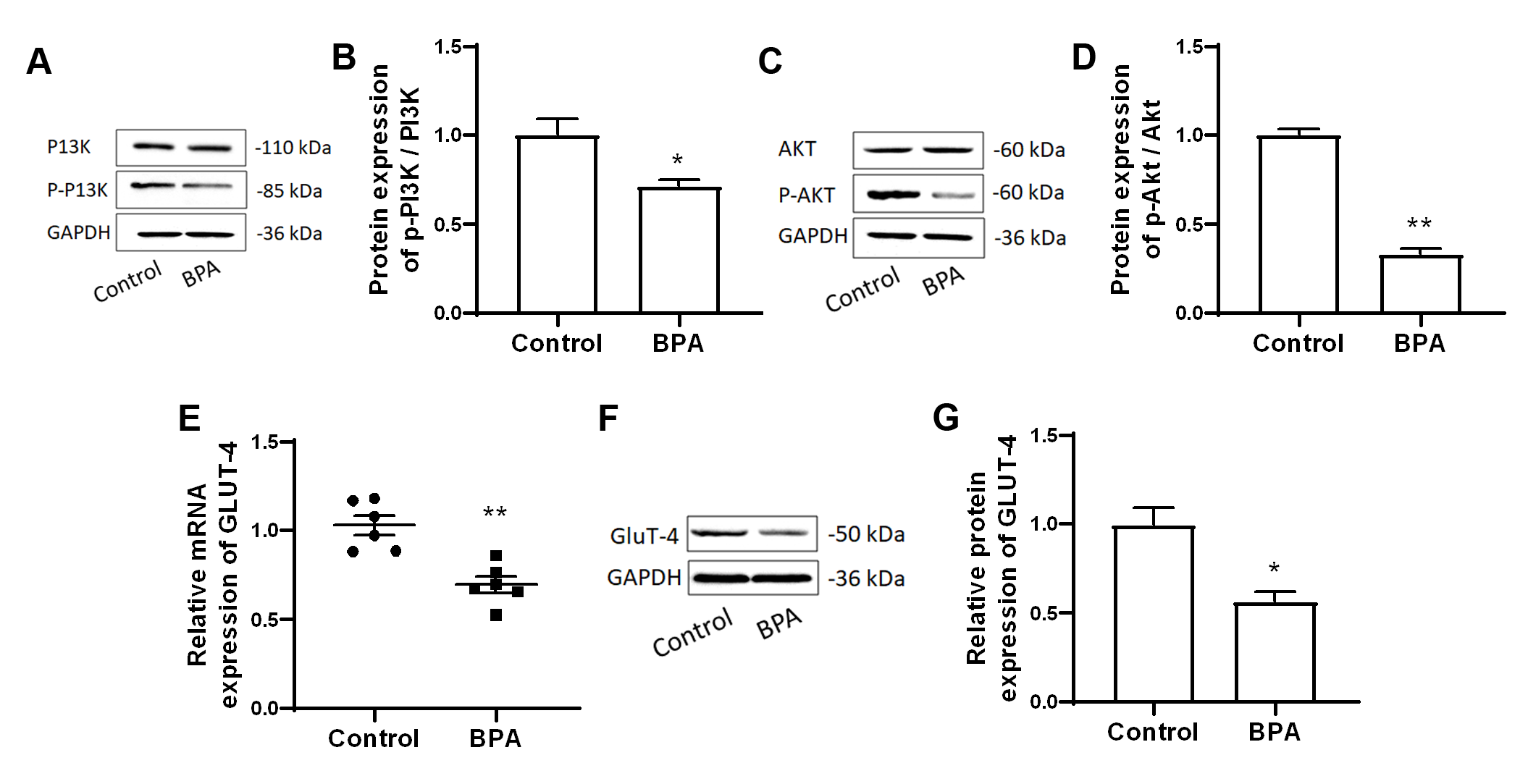


**Supplemental Fig.3. The effects of BPA in PI3K/Akt signaling pathway.**

Protein expression of (A, B) *PI3K/p-PI3K* and (C, D) *Akt/p-Akt*, as well as (E, F, G) both mRNA and protein expression levels of *GLUT-4* were determined in the liver of male offspring using qRT-PCR and immunoblotting. **p < 0.01, indicate significant differences when compared to respective controls.


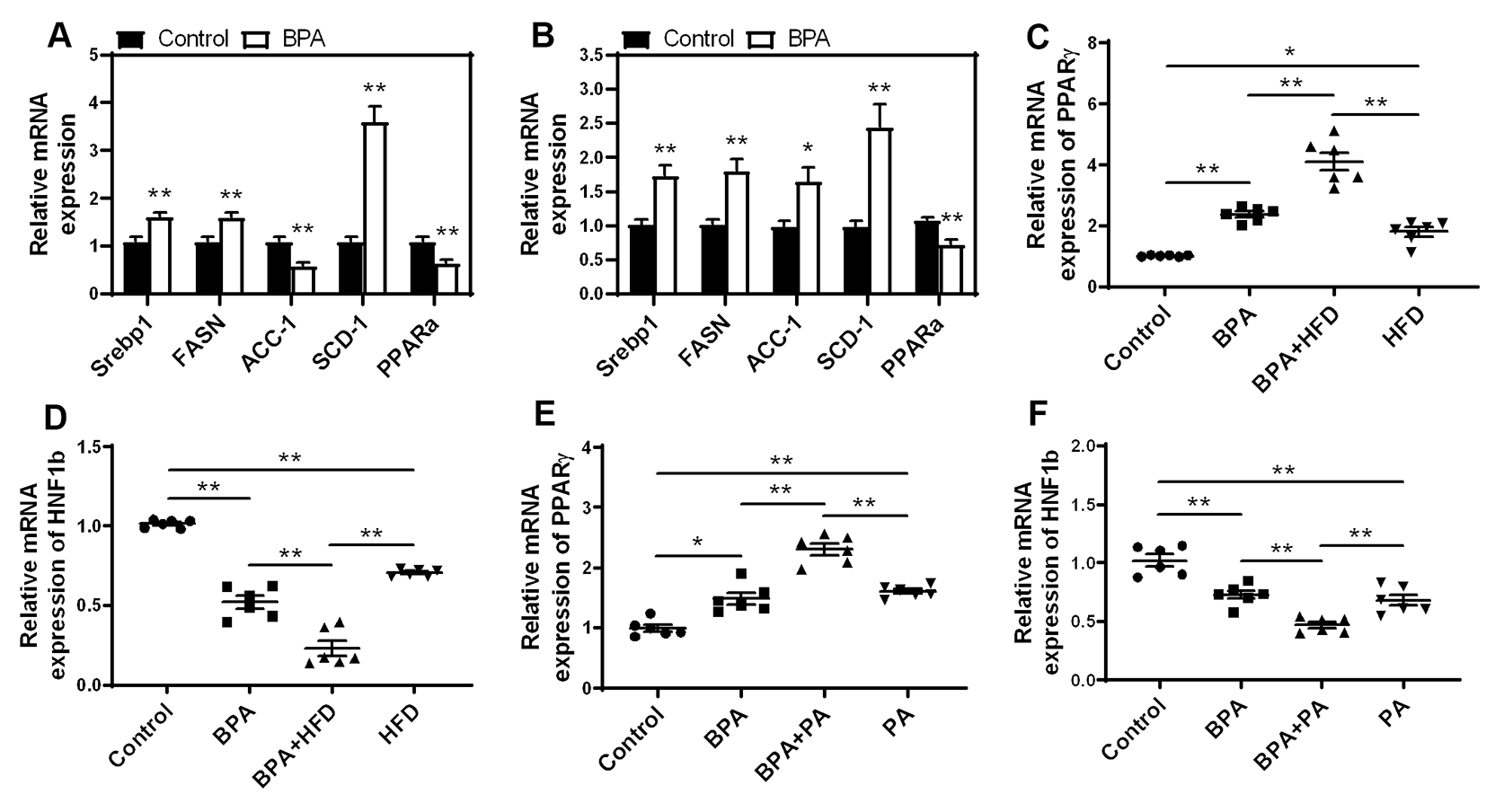


**Supplemental Fig.4. The effects of BPA on the expression of key regulators involved in lipid metabolism.**

(A, B) Genes involved in the regulation of lipid metabolism were determined in male mouse livers and L02 cells. These genes include *Srebp1, FASN, ACC-1, SCD-1, PPARα* *p < 0.05, **p < 0.01, compared with control. Both mRNA and protein expression levels of *PPARγ* and *HNF1b* were identified both *in vivo* in the livers of (C, D, E, F) male mice and *in vitro* in (G, H, I, J) L02 cells using qRT-PCR and immunoblotting, *p < 0.05, **p < 0.01, indicate significant differences when compared between the two groups.


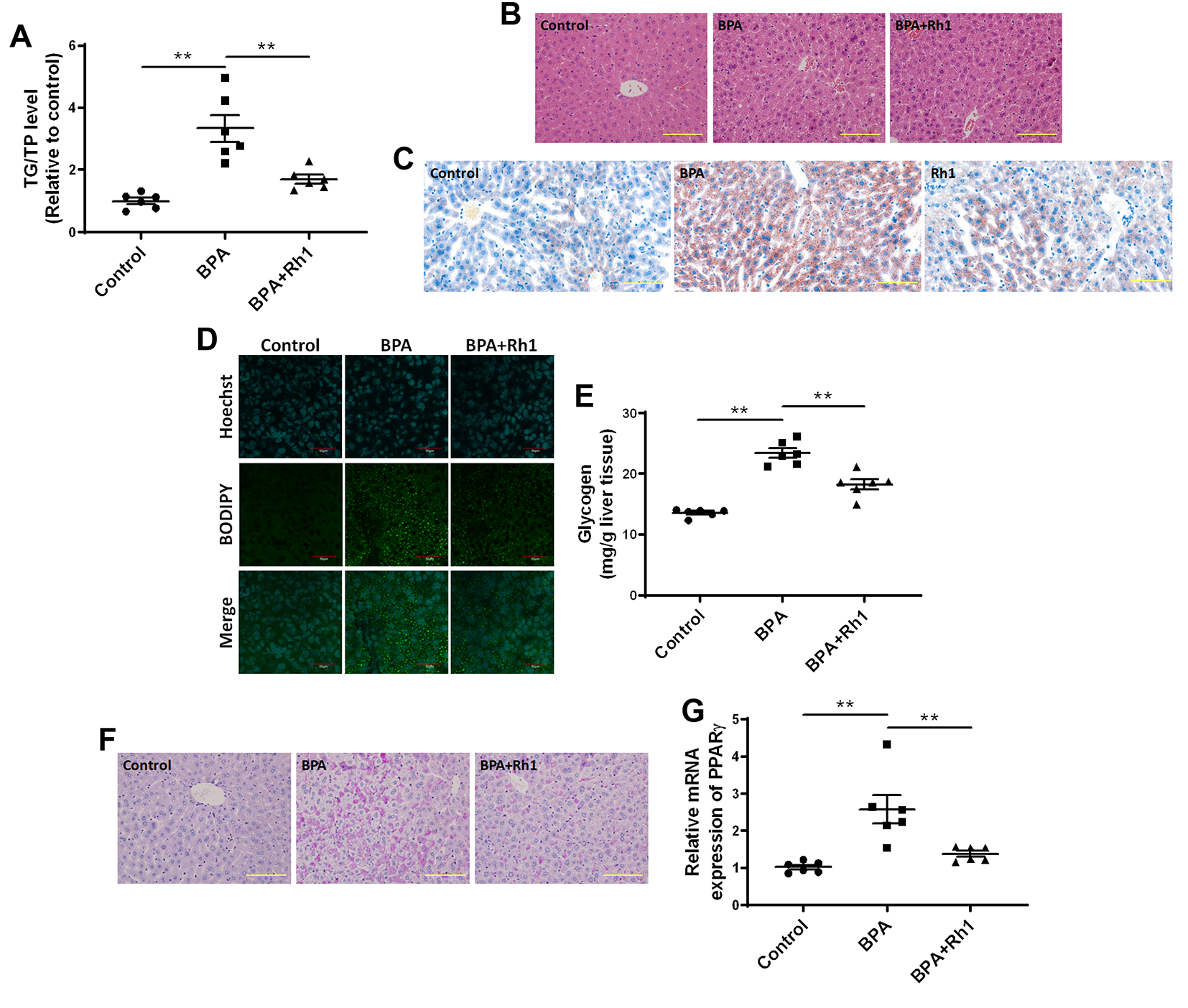


**Supplemental Fig.5. The upregulation of PPARγ is involved in BPA-induced effects.**

(A) Determination of Liver TG contents in adult male mice. (B) H&E staining of liver tissue was completed to the valuation of lipid accumulation. Frozen sections were prepared and stained with (C) Oil Red O and (D) BODIPY to observe lipid droplets. (E) Glycogen content was detected and (F) PSA staining was used to display glycogen in liver tissues. Size bar = 50μm. (G, H, I, J) The mRNA and protein expression levels of PPARγ and HNF1b were identified using qRT-PCR and immunoblotting. **p < 0.01, indicate significant differences when compared between the two groups.


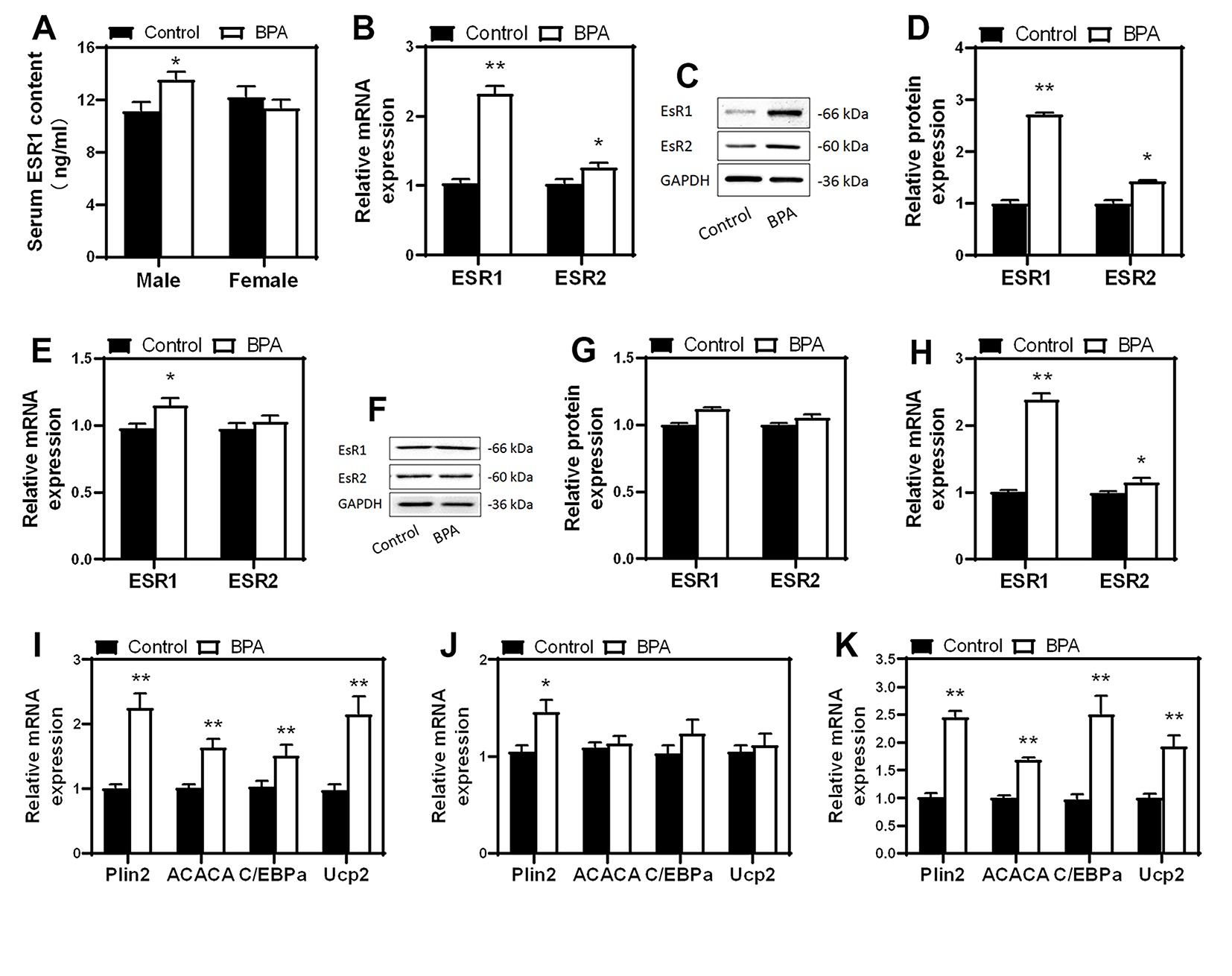


**Supplemental Fig.6. *In Vivo* and *in Vitro e*ffects of BPA on estrogen-related regulators.**

(A) Determination of serum ESR1 contents. Both mRNA and protein expression patterns of ESR1 and ESR2 were determined in the liver tissues of male offspring (B, C, D) and female offspring (E, F, G) mice using qRT-PCR and immunoblotting. (H) mRNA expression of *ESR1* and *ESR2* were determined in L02 liver cells. A variety of genes involved in estrogen regulation were identified in the liver of (I) male and (J) female offspring and (K) L02 liver cells using qRT-PCR, including the genes *Plin2,* *ACACA*, *C/EBPα*, *Ucp2*. *p < 0.05, **p < 0.01, indicate significant differences when compared to respective controls.
